# Exploration of the Out‐of‐Phase Phenomenon in Shake Flasks by CFD Calculations of Volumetric Power Input, k_L_a Value and Shear Rate at Elevated Viscosity

**DOI:** 10.1002/bit.28892

**Published:** 2024-11-30

**Authors:** Carl Dinter, Andreas Gumprecht, Matthias Alexander Menze, Amizon Azizan, Sven Hansen, Jochen Büchs

**Affiliations:** ^1^ AVT – Biochemical Engineering RWTH Aachen University Aachen North Rhine‐Westphalia Germany; ^2^ Evonik Operations GmbH Hanau‐Wolfgang Hesse Germany; ^3^ School of Chemical Engineering, College of Engineering Universiti Teknologi MARA Shah Alam Selangor Malaysia; ^4^ Evonik Operations GmbH Halle (Westf.) North Rhine Westphalia Germany

**Keywords:** CFD, k_L_a value, out‐of‐phase phenomenon, shake flask, shear rate, viscosity, volumetric power input

## Abstract

Culture broth with secreted macromolecules and culture broth of filamentous fungi showing disperse growth exhibit elevated viscosity, usually with shear‐thinning flow behavior. High viscosity, however, poses a serious challenge in the production and research of these compounds and organisms. It commonly causes insufficient mixing and oxygen transfer in large‐ and small‐scale bioreactors. Computational Fluid dynamics (CFD) has been proven to be a valuable tool for the computation of important bioprocess parameters. The published literature for small‐scale shaken bioreactors, especially shake flasks, however, almost exclusively focuses on water‐like viscosity. In this paper, a previously published CFD model for 250 mL shake flasks was used to simulate experiments at high viscosities of up to 100 mPa·s. Compared to experimental data, the CFD model accurately predicted the liquid distribution and computed the volumetric power input with a deviation of less than 7% and the k_L_a value within a factor of two, compared to the k_L_a correlation from Henzler and Schedel. Furthermore, a novel approach to compute the shear rate was tested. Lastly, new insights into the out‐of‐phase phenomenon were gained. The presented data confirms the usefulness of the already established critical phase numbers of 0.91 and 1.26, while underlying the fundamentally smooth transition from in‐phase to out‐of‐phase operating conditions.

## Graphical abstract

Next‐generation cell‐based therapies require enhanced safety and spatiotemporal control, which can be ensured through the incorporation of synthetic gene switches. This review details recent developments in synthetic gene switches responsive to different molecular inputs and acting at the transcriptional, translational, and post‐translational levels. It also explores current perspectives of cell‐based therapies and the challenges facing clinical translation.

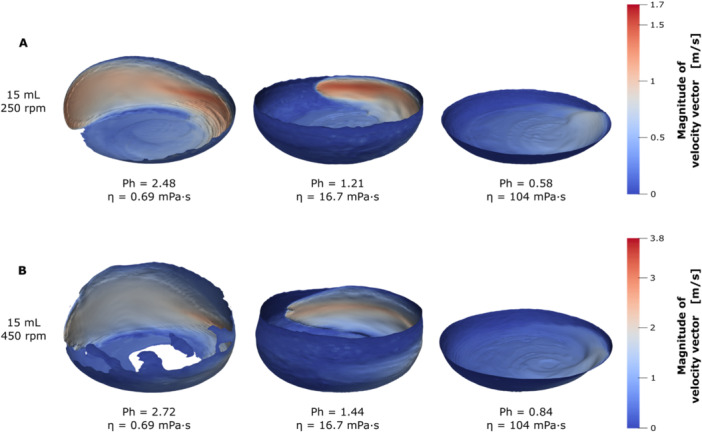

## 1 Introduction

Due to their useful rheological properties, biopolymers are used in medicine for drug delivery and drug release systems, wound dressing and injections for joint health and as additives in the food and cosmetic industry (Serra et al. 2023; Seviour et al. 2011; Sparviero et al. 2023). Filamentous fungi are, similarly, applied industrially for the production of useful chemical compounds, including organic acids, antibiotics, enzymes like glucoamylases and as meat substitutes (Füting et al. 2021). Culture broth with secreted macromolecules and culture broth of filamentous fungi exhibiting disperse growth are virtually always viscous, non‐Newtonian fluids, showing pronounced shear‐thinning behavior, that is, a reduction in viscosity with increasing shear rates. The increasing viscosity during fermentation, however, leads to a lot of problems, including diminished mixing efficiency and insufficient oxygen supply (Peter et al. 2004; Seviour et al. 2011). Researchers have extensively investigated the precise influence of viscosity in shake flask experiments. Its influence on volumetric power input, oxygen transfer, shear rate and k_L_a value have been measured and modeled.

Most notably, viscosity is one of the major contributors to the so‐called out‐of‐phase phenomenon, described by Büchs et al. (2000a, 2000b). The out‐of‐phase phenomenon essentially describes a situation where the centrifugal force caused by the orbital shaking motion of the shake flask is insufficient to accelerate the bulk liquid. Under in‐phase operating conditions, the bulk liquid follows the shaking motion of the shaker, whereas under out‐of‐phase operating conditions a complete collapse of the bulk liquid with uncoordinated liquid movement can be observed. Low shaking frequency, low filling volume, small shaking diameters and, importantly, high viscosity all contribute to out‐of‐phase operating conditions. Büchs et al. (2000a, 2000b) suggested the dimensionless phase number as an indicator for the degree, to which the liquid is out‐of‐phase with the shaking motion. They experimentally determined volumetric power inputs for six different sizes of shake flasks from 100 to 2000 mL for a variety of operating conditions. Based on their experiments, they found a critical phase number of 1.26, below which the out‐of‐phase phenomenon predominates (Büchs et al. 2000b). At in‐phase operating conditions, the volumetric power input increases continuously with increasing viscosity, but at out‐of‐phase operating conditions, the volumetric power input becomes far less predictable and stagnates or sometimes even declines. Büchs et al. (2000b) provide a correlation of the Reynolds number and modified Newton number for in‐phase operating conditions, enabling the calculation of volumetric power inputs. Azizan et al. (2019) further investigated the out‐of‐phase phenomenon and used experimental liquid distributions in shake flasks to determine a new critical phase number of 0.91.

A comprehensive description of oxygen transfer in shake flasks is given by Maier, Losen and Büchs (2004). In their so‐called “two sub‐reactor model”, the oxygen transfer into the rotating bulk liquid is the first sub‐reactor and can be calculated with the models by Kawase and Moo‐young (1990) or by Gnielinski (1975). The second sub‐reactor is the liquid film, which is formed and left behind on the flask wall by the rotating bulk liquid. The oxygen transfer into this liquid film is modeled with Higbie's penetration theory. An increase in viscosity significantly lowers the diffusion coefficient in the bulk liquid and film. It, however, also leads to thicker liquid films on the shake flask wall. Giese et al. (2014) found that small increases in viscosity lead to an increase in the maximum oxygen transfer capacity, despite the reduction of the oxygen diffusion coefficient in the liquid. The increasing liquid film thickness, leading to an increased capacity to store oxygen in the film, explains this effect. At a specific film thickness, however, the oxygen transfer in the film is no longer limited by the capacity to store oxygen in the film. Rather, the duration until the bulk liquid rolls over the liquid film again, which depends on the shaking frequency of the shaking table, limits the oxygen transfer. The liquid film no longer reaches oxygen saturation during the revolution, hence, the reduction of the oxygen diffusion coefficient, with increasing viscosity, outweighs the benefits of an increased capacity to store oxygen. The maximal oxygen transfer capacity is reached at roughly 10–20 mPa·s in shake flasks. Besides the model by Maier, Losen and Büchs (2004), multiple attempts to correlate the k_L_a value to the operating conditions in shake flasks have been published (Henzler and Schedel 1991; Nikakhtari and Hill 2008; Van Suijdam, Kossen, and Joha 1978; Veglio, Beolchini, and Ubaldini 1998; Veljković et al. 1995). However, to our knowledge, only Henzler and Schedel included the viscosity in their correlation by fitting to experimental data for a variety of viscosities.

As discussed, biopolymers exhibit non‐Newtonian properties, requiring the estimation of shear rates to accurately determine the effective viscosity in shake flask experiments. Giese et al. (2014) developed a correlation for effective shear rates in shake flasks for in‐phase operating conditions with shear‐thinning fluids. They used a power law approach to model the shear‐thinning behavior.

Computational fluid dynamics (CFD) has proven to be an extremely valuable tool for the computation of common process performance indicators, such as volumetric power input, shear stress and k_L_a value (X. Li et al. 2018; Santos‐Moreau, Lopes, and Fonte 2019; Wutz et al. 2016). Although CFD is applied much more commonly for large‐scale stirred tank reactors, some research for CFD in small‐scale shaken bioreactors, including shake flasks (C. Li et al. 2013; Liu et al. 2016; Mehmood et al. 2010; Zhang et al. 2005), microtiter plates (He et al. 2023; Lu et al. 2021; Montes‐Serrano et al. 2022; Wutz et al. 2018; Zhang et al. 2008), and other small cylindrical vessels (Discacciati et al. 2013; Zhu et al. 2018, 2022) has been published. This research, however, is almost exclusively focused on a waterlike viscosity of about 1 mPa·s. The only exceptions are the works by Mehmood et al. (2010) for up to 3 mPa·s in shake flasks, by Discacciati et al. (2013) for extremely high viscosity of 1000 mPa·s in small cylinders and our previous publication for up to 16 mPa·s in shake flasks (Dinter et al. 2024). This paper expands on our previous publication, simulating up to 100 mPa·s in 250 mL shake flasks, reaching out‐of‐phase operating conditions. The simulations are compared to experimentally determined liquid distributions from Azizan and Büchs (2017), experimentally determined volumetric power inputs, k_L_a values, calculated with the correlation by Henzler and Schedel (1991) and shear rates, calculated with the correlation by Giese et al. (2014).

## 2 Materials and Methods

The interFoam solver from OpenFOAM v9 was used to solve the Navier‐Stokes equation for the two‐phase system in shake flasks. Our previous publication (Dinter et al. 2024) provides a more detailed description of our CFD model, the interFoam solver, the approximation of the shaking motion, modeled flask geometry and mesh generation. The supplement provides an example of a meshed 250 mL shake flask (Supporting Information: Figure S1). The next subchapters provide a brief recap of the calculation of volumetric power inputs from the CFD model and by the correlation from Büchs et al. (2000b). Furthermore, the computation of the k_L_a value and shear rate from the CFD model are explained.

### 2.1 Calculation of Volumetric Power Input From CFD Simulations

The volumetric power input is proportional to the energy dissipation rate and can be described as:

(1) $$\frac{P}{V_{L}}=\varepsilon \;\cdot \;\rho$$

where *P*, *V_L_*, *ε*, and *ρ* are the power input, the liquid volume, the energy dissipation rate and density. The energy dissipation rate can be calculated as follows:

(2) $$\varepsilon =\frac{\int_{V_{L}}\left(\frac{\eta }{\rho }\;\cdot \;||S|{|}^{2}+\beta^{*}\omega k\right)\;\cdot \;dV_{L}}{V_{L}},$$

where *η*, ||**S** ||, *ω*, and *k* are the viscosity, a scalar measure of the strain rate tensor, specific energy dissipation rate and turbulent kinetic energy, respectively. The specific energy dissipation rate and turbulent kinetic energy are taken directly from the k‐ω SST turbulence model, which is described in our previous publication (Dinter et al. 2024). The k‐omega SST turbulence model is an eddy viscosity model and implements transport equations for the turbulent kinetic energy and specific rate of dissipation. The specific rate of dissipation transport equation of the k‐ω SST model is a blend of the transport equations from the k‐ε and k‐ω turbulence models. It, thereby, combines the good prediction of free shear stream of the k‐epsilon model with the better performance near walls of the k‐ω model. β* is a model constant of the k‐omega SST model and is given as 0.09. The strain rate tensor can be expressed as the symmetric part of the Helmholtz tensor decomposition of the velocity gradient tensor ∇**U** (Wu et al. 2020), which leads to:

(3) $$||S||=\sqrt{2S:S}=\sqrt{2\;\cdot \;\left[{\left(\frac{\partial u}{\partial x}\right)}^{2}+{\left(\frac{\partial v}{\partial y}\right)}^{2}+{\left(\frac{\partial w}{\partial z}\right)}^{2}\right]+{\left(\frac{\partial u}{\partial y}+\frac{\partial v}{\partial x}\right)}^{2}+{\left(\frac{\partial v}{\partial z}+\frac{\partial w}{\partial y}\right)}^{2}+{\left(\frac{\partial u}{\partial z}+\frac{\partial w}{\partial x}\right)}^{2}}$$

as a scalar measure of the strain rate tensor, where *u*, *v*, and *w* are the velocity components in direction of the Cartesian coordinates *x*, *y*, and *z*.

### 2.2 Calculation of the Phase Number

The phase number is calculated according to Büchs et al. (2000b) as follows:

(4) $$Ph=\frac{d_{0}}{d}\;\cdot \;(1+3\;\cdot \;\log_{10}\left({\text{Re}}_{\mathrm{film}}\right)),$$

where *Ph* and Re_film_ are the phase number and Reynolds film number. The Reynolds film number is given as:

(5) $${\text{Re}}_{\mathrm{film}}=\text{Re}\;\cdot \;\frac{\pi }{2}\;\cdot \;{\left(1-\sqrt{1-\frac{4}{\pi }\;\cdot \;{\left(\frac{{V_{L}}^{\frac{1}{3}}}{d}\right)}^{2}}\right)}^{2}.$$

### 2.3 Volumetric Power Input Correlation

Volumetric power inputs for shake flasks can be calculated from the correlation of the Reynolds number (Re) and modified Newton number (*Neʹ*) by Büchs et al. (2000a, 2000b):

(6) $$Ne^{\prime }=70\;\cdot \;{\text{Re}}^{-1}+25\;\cdot \;{\text{Re}}^{-0.6}+1.5\;\cdot \;{\text{Re}}^{-0.2},$$

where the Reynolds number [‐] and modified Newton number [‐] are given as follows:

(7) $$Ne^{\prime }=\frac{P}{\rho \;\cdot \;n^{3}\;\cdot \;d^{4}\;\cdot \;V_{L}^{\frac{1}{3}}},$$

(8) $$\text{Re}=\frac{\rho \;\cdot \;n\;\cdot \;d^{2}}{\eta },$$

where *n*, *d*, and *V_L_* are the shaking frequency, the largest inner flask diameter and the filling volume. Büchs et al. (2000a, 2000b) derived this correlation of Reynolds and modified Newton number from volumetric power inputs determined experimentally at a variety of shaking conditions for 100–2000 mL shake flasks. The derived correlation includes only experiments, which were deemed to be in‐phase by Büchs et al., based on a critical phase number of 1.26.

### 2.4 Diffusion Coefficient for Small Molecules

The diffusion coefficient is necessary to calculate the k_L_a value. For the calculation of the diffusion coefficient D_L_, the Stokes–Einstein Gierer–Wiertz estimation is used (Evans et al. 2018). It is an extension of the Stokes–Einstein equation, which is the ratio of thermal energy and friction forces and can be given for spherical molecules as follows:

(9) $$D_{L}=\frac{k_{B}\;\cdot \;T}{6\;\cdot \;\pi \;\cdot \;\eta \;\cdot \;r_{M}},$$

where *k_B_* is the Boltzmann constant, *T* the temperature, *η* the dynamic viscosity, and *r_M_* the molecule radius. This equation follows the assumption of a particle randomly moving through a continuous medium. However, for the traversal of small molecules, that is gases, the assumption of a continuous medium does not hold. Instead, the physical interactions of traversing small molecule and molecules of the liquid phase, moving randomly in space, should be considered. The interaction of molecules can be expressed as a modification of the friction forces in the standard Stokes–Einstein equation. Gierer and Wirtz modeled this behavior as the correction factor *f*_*GW*_ (Evans et al. 2018):

(10) $$f_{\mathrm{GW}}={\left(\frac{3\beta }{2}+\frac{1}{1+\beta }\right)}^{-1},$$

where *β* is the ratio of radii of the solute and solvent. Applying the assumption of hard spheres, Evans et al. (2018) estimate the radii of solute and solvent based on the molecular weights as follows:

(11) $$r_{M}=\sqrt[{3}]{\frac{3\;\cdot \;\mathrm{MW}}{4\;\cdot \;\pi \;\cdot \;\rho_{\mathrm{eff}}\;\cdot \;N_{A}}},$$

where *MW* is the molecular weight, *ρ_eff_* the effective density and *N_A_* Avogadro's constant. Evans et al. (2018) give an effective density of 619 kg/m^−3^, which they obtained by fitting measurement data for 109 combinations of 44 solutes and five solvents. Combining Equations ((9), (10), (11)) yields the following equation for the diffusion coefficient:

(12) $$D_{L}=\frac{k_{B}\;\cdot \;T\;\cdot \;\left(\frac{3\;\cdot \;\sqrt[{3}]{\frac{{\mathrm{MW}}_{\mathrm{solute}}}{{\mathrm{MW}}_{\mathrm{solvent}}}}}{2}+\frac{1}{1+\sqrt[{3}]{\frac{{\mathrm{MW}}_{\mathrm{solute}}}{{\mathrm{MW}}_{\mathrm{solvent}}}}}\right)}{6\;\cdot \;\pi \;\cdot \;\eta \;\cdot \;\sqrt[{3}]{\frac{3\;\cdot \;{\mathrm{MW}}_{\mathrm{solute}}}{4\;\cdot \;\pi \;\cdot \;\rho_{\mathrm{eff}}\;\cdot \;N_{A}}}}.$$

### 2.5 Calculation of the k_L_a Value From CFD Simulations

The k_L_a value consists of the mass transfer coefficient k_L_ and the specific surface area a. In this work the eddy cell model by Lamont and Scott (1970) was used to calculate the k_L_ value for the CFD simulations. Similar to the calculation of the volumetric power input, the model is based on the energy dissipation rate (see Equation 2) and is given as follows:

(13) $$k_{L}=0.4\;\cdot \;\sqrt{D_{L}}{\left(\frac{\varepsilon }{\nu }\right)}^{\frac{1}{4}},$$

where *D_L_* is the diffusion coefficient (see Equation 12), *ε* the energy dissipation rate (see Equation 2) and *ν* the kinematic viscosity. The specific surface area a of a mesh cell *i* in the CFD model can be approximated as the magnitude of the gradient of the volume fraction *α* of that cell (Soh, Yeoh, and Timchenko 2016; Wutz et al. 2018):

(14) $$a_{i}=|{\nabla \alpha }_{i}|.$$

The k_L_a value was then calculated on a per cell basis and integrated over the entirety of the CFD model as follows:

(15) $$k_{L}a=\frac{\int_{V}k_{L,i}\;\cdot \;a_{i}\;\cdot \;\mathrm{dV}}{V}.$$

### 2.6 Correlation for k_L_a Values in Shake Flask Experiments

The correlation from Henzler and Schedel (1991) describes the *k_L_a* value in shake flask experiments as follows:

(16) $$k_{L}a=0.5\;\cdot \;d^{\left(\frac{73}{36}\right)}\;\cdot \;n\;\cdot \;d_{0}^{\left(\frac{1}{4}\right)}\;\cdot \;V_{L}^{\left(-\frac{8}{9}\right)}\;\cdot \;\nu^{\left(-\frac{13}{54}\right)}\;\cdot \;g^{\left(-\frac{7}{54}\right)}\;\cdot \;D_{L}^{\left(\frac{1}{2}\right)},$$

where *d*, *n*, *d*_0_, *V_L_*, *ν*, *g*, and *D_L_* are the largest inner flask diameter, shaking frequency, shaking diameter, filling volume, kinematic viscosity, gravitational acceleration and diffusion coefficient.

### 2.7 Approximation of the Shear‐Thinning Behavior as a Power‐Law Fluid

The simplest and most common approach to approximate the non‐Newtonian flow behavior of shear‐thinning fluids is the power‐law model, also routinely referred to as the Ostwald‐de Waele relationship (Ostwald 1929). The power‐law model can be given as the relationship of shear stress and shear rate as follows:

(17) $$\tau =K\;\cdot \;\gamma^{m},$$

where *τ*, *γ*, *K*, and *m* are the shear stress, shear rate, flow consistency index and flow behavior index. The power‐law model can also be given as the relationship of viscosity and shear rate as follows:

(18) $$\eta_{\mathrm{eff}}=K\;\cdot \;\gamma^{m-1}$$

where *η*_*eff*_ is the effective viscosity for the given shear rate.

### 2.8 Calculation of the Shear Rate From CFD Simulations

The shear rate for CFD simulations was calculated in two ways. First, as the square root of the energy dissipation. This is a common approach, utilized in OpenFOAM (OpenFOAM v9 Github 2023), which is given as follows:

(19) $$\gamma_{i}=\sqrt{∥S∥},$$

where *γ_i_* and ||**S**|| are the shear rate in mesh cell *i* and the scalar measure of the strain rate tensor. The strain rate tensor is defined as the symmetric part of the gradient of the velocity vector field. The symmetric part describes the rate of shearing and stretching in the velocity field. The antisymmetric part, called vorticity tensor or spin tensor, describes only rotational movement. The second approach uses the estimation of average shear stress based on the eigenvalues of the strain rate tensor. Soos et al. (2010) give the equation of average shear stress as follows:

(20) $$\tau_{L}=2.5\;\cdot \;\eta \;\cdot \;\lambda_{1}\left(S\right),$$

where *τ_L_* is the average shear stress, *η* the dynamic viscosity and *λ*_1_(**S**) the largest, positive eigenvalue of the strain rate tensor **S**. Under the assumption of Newtonian flow behavior, the shear rate for a mesh cell *i* can be easily calculated from the shear stress, leading to the following equation:

(21) $$\gamma_{i}=2.5\;\cdot \;\lambda_{1}\left(S_{i}\right),$$

where *γ_i_* and **S**_*i*_ are the shear rate and strain rate tensor in mesh cell *i*. For both approaches the shear rates per cell are then integrated over the liquid volume of the CFD model as follows:

(22) $$\gamma =\frac{\int_{V_{L}}\gamma_{i}\;\cdot \;\alpha_{i}\;\cdot \;dV_{L}}{V_{L}}.$$

Under the assumption of non‐Newtonian flow, the equation for shear stress (Equation 20) cannot directly be transformed to an estimation of the shear rate (Equation 21). Instead, the shear stress would need to be calculated for an intermediate viscosity, depending on some intermediate shear rate as follows:

(23) $$\tau_{L,\mathrm{inter}}=2.5\;\cdot \;\eta_{\mathrm{inter}}\left(\gamma_{\mathrm{inter}}\right)\;\cdot \;\lambda_{1}\left(S\right)$$

where *τ*_*L,inter*_, *η*_*inter*_, and *γ*_*inter*_ are the intermediate shear stress, viscosity and shear rate. An approximation of the non‐Newtonian fluid with, for example, a power‐law approach (see Equation 17) would, after rearranging, enable the calculation of the shear rate as follows:

(24) $$\gamma_{\mathrm{inter},\mathrm{new}}={\left(\frac{\tau_{L,\mathrm{inter}}}{K}\right)}^{\frac{1}{m}},$$

where *K* and *m* are the flow consistency index and flow behavior index from the power‐law. Hence, the calculation of the shear rate would have to be performed iteratively in the CFD, when non‐Newtonian fluids are simulated.

### 2.9 Correlation for Effective Shear Rates in Shake Flask Experiments

The correlation for effective shear rates by Giese et al. (2014) is given as follows:

(25) $$\gamma =2.06^{\frac{1}{m+1}}\;\cdot \;{\left(\frac{P}{V_{L}\;\cdot \;K}\right)}^{\frac{1}{m+1}}\;\cdot \;{\left(\frac{{V_{L}}^{\frac{1}{3}}}{d}\right)}^{\frac{-0.331}{m+1}}.$$

## 3 Results and Discussion

### 3.1 Liquid Contact Lines

**Figure 1:**
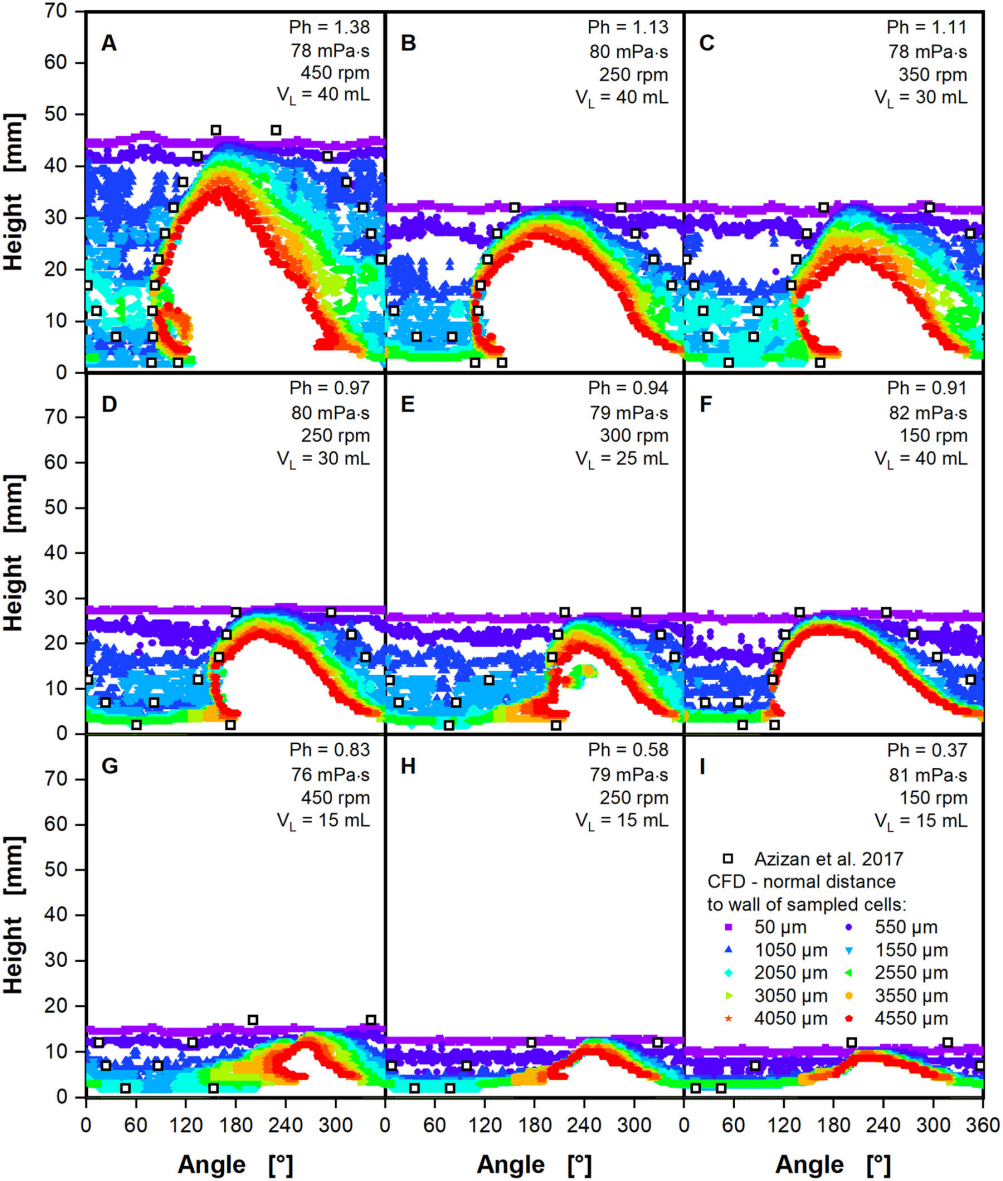
Liquid contact lines extracted from the computational fluid dynamics (CFD) model, compared to experimental data (open black squares) by Azizan et al. (2019) and Azizan and Büchs (2017). The liquid contact lines are shown in mm, viewed from the center of the shake flask, rotating around the z‐axis (see Supporting Information: Figure S2). To exclude the liquid film, liquid contact lines from the CFD simulations were extracted at multiple distances from 50 to 4550 µm normal to the shake flask wall (as indicated in Supporting Information: Figure S1A). Simulated conditions are ordered from highest (A) to lowest (I) phase number (Equation 4). The polyvinylpyrrolidone, used by Azizan et al. (2019) and Azizan and Büchs (2017) shows shear‐thinning. Hence, Newtonian viscosities for CFD simulations were determined by calculating the effective shear rate according to Giese et al. (2014) (Equation 25) and then estimating the viscosity with the power law for non‐Newtonian fluids (Equation 18). Simulated conditions: Shaking diameter (*d*_0_) = 25 mm, surface tension (*σ*) = 70 mN/m, contact angle (*θ*) = 20°, temperature (*T*) = 20°C, consistency factor (*K*) = 104 mPa·s and flow behavior index (*m*) = 0.956 according to Azizan et al. (2019) and Azizan and Büchs (2017).

As a first test of CFD model performance at high viscosity, the liquid distribution was analyzed, which can be regarded as a freeze frame of the rotating fluid flow. Liquid contact lines (see Supporting Information: Figure S2) at the intersection of shake flask wall, liquid phase and gaseous phase were extracted from the CFD model and compared to experimentally determined liquid contact lines from Azizan et al. (2017, 2019). Azizan et al. (2017, 2019) determined the liquid contact lines based on noninvasive fluorescence measurements. Fluorescence measurements enable the distinction between water and air in front of the sensor. They measurements were performed at 14 distinct heights, each 5 mm apart during the shaking motion. Figure 1 depicts a comparison of the liquid contact lines at about 80 mPa·s, for a variety of shaking conditions, including shaking frequencies between 150 and 450 rpm and filling volumes between 15 and 40 mL. CFD simulations were conducted for Newtonian fluids, i.e., at a constant viscosity. The polyvinylpyrrolidone, used by Azizan et al. (2017, 2019) for the evaluation of experimental liquid contact lines, however, shows slight shear‐thinning behavior, i.e., a reduction in viscosity with increasing shear rates. Before performing the CFD simulations, effective shear rates for the given shaking conditions were calculated according to Giese et al. (2014) and used to determine constant viscosities for the simulations. This leads to small variations in the viscosity around 80 mPa·s for the CFD simulations in Figure 1. The simulations in Figure 1 are ordered from highest to lowest phase number, crossing both the critical phase number by Büchs et al. (2000b) of 1.26 and by Azizan et al. (2019) of 0.91.

Due to the occurrence of liquid films in shake flasks, liquid contact lines were not only extracted directly at the flask wall, but at multiple, iteratively increasing normal distances to the shake flask wall (see Supporting Information: Figure S1). The smallest, considered normal distance of 50 µm ensures the extraction of the liquid contact line from the first wall layer of the generated mesh. This first layer of cells in the mesh has a width of 140 µm. This normal distance is increased by 500 µm in each iteration, until 4550 µm are reached. For the lowest normal distance to the flask wall of 50 µm (violet square), a constant liquid height over the entire flask circumference can be observed for every depicted case, deviating only marginally from the maximal liquid height of the experimentally determined liquid contact lines. Liquid contact lines at the larger normal distances from 1550 µm onward (light blue, upside down triangles) reveal the rotating bulk liquid for all depicted cases in Figure 1. The liquid film thickness in the CFD increases from 50 µm (violet square) at the maximal liquid height to 2050 µm (blue‐green diamonds) at the base of the shake flask, averaging around 1050 µm (blue triangles). Experimentally evaluated liquid films from Hermann (2002), reproduced in Dinter et al. (2024), confirm a film thickness of roughly 1000 µm at 100 mPa·s.

At the normal distances of 1550 µm (light blue, upside down triangles) and larger, the leading edge of the rotating bulk liquid (left flank) is clearly visible in the CFD simulations in Figure 1A–F. In the leading edge the data points for the offsets from 1550 µm onward are stacked on top of each other, signifying a large difference in the liquid thickness between liquid film and bulk liquid. For the cases in Figure 1A–F the leading edge in the CFD simulation agrees well with the experimentally determined liquid contact lines, deviating a few degrees at most. In the tail region, the data points for all offsets are spread out over a large section of the flask wall, indicating a smooth transition from bulk liquid to liquid film. This complicates a clear distinction into the tail region of the bulk liquid and liquid film, affecting both CFD and experimental values. Yet, as explained above, the liquid film should show a thickness of around 1000 µm. Hence, the edge of the tail region can be roughly defined as the change from the offset of 1550 µm (light blue, upside down triangles) to 1050 µm (blue triangles). This region agrees well with the experimentally determined tail of the rotating bulk liquid in Figure 1A–F. In Figure 1G–I the phase numbers are significantly below the critical phase number by Azizan et al. (2019) of 0.91. Under this critical phase number, a complete collapse of the rotating bulk liquid has been described, which is confirmed by the liquid distribution from CFD and experiments in Figure 1G–I. The maximal liquid height is greatly reduced, compared to the cases in Figure 1A–F, showing only a small wave flowing on the bottom of the shake flask. In summary, the liquid contact lines from the CFD model fit well to the experimental data from Azizan et al. (2017, 2019).

### 3.2 Volumetric Power Inputs

**Figure 2:**
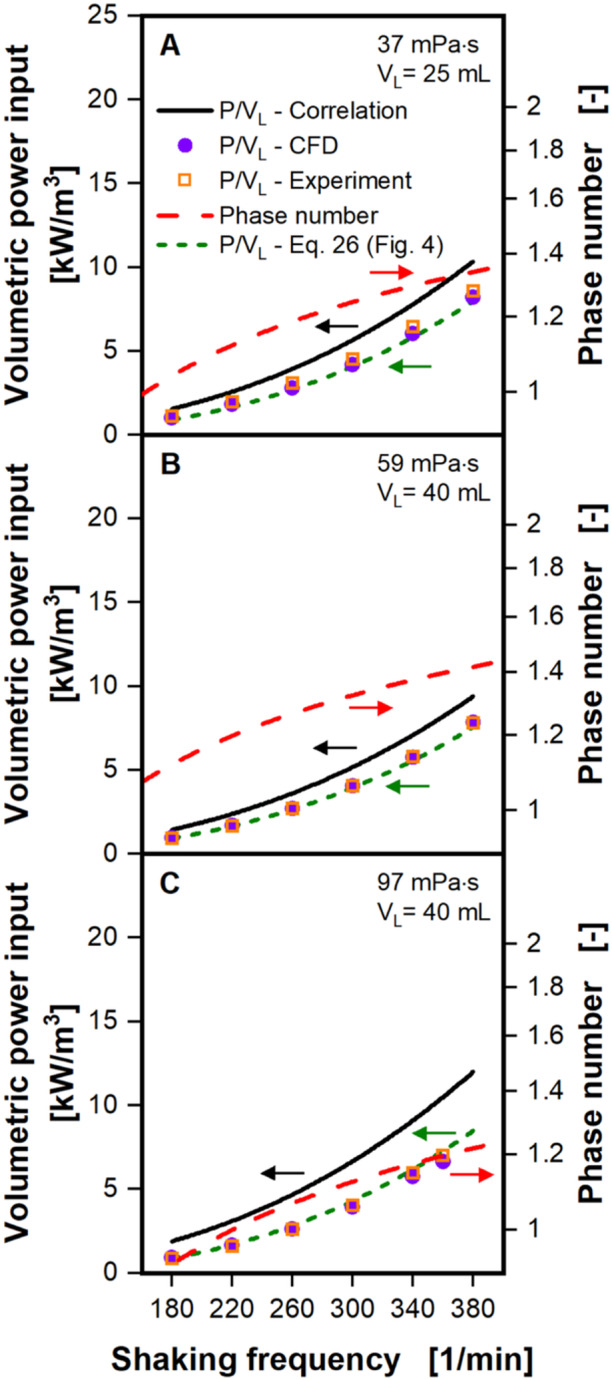
Volumetric power input as a function of shaking frequency at elevated viscosity. Volumetric power inputs for viscosities of 37 mPa·s at V_L_ = 25 mL filling volume and 59 and 97 mPa·s at V_L_ = 40 mL filling volume are shown in (A), (B), and (C), respectively. Values derived from the CFD model, experimentally determined values and values calculated with the power input correlation (Equation 6), developed for in‐phase operating conditions by Büchs et al. (2000b, 2000a), are compared. Phase numbers are calculated according to Büchs et al. (2000b) (see Equation 4) and are depicted on a logarithmic scale. The green dashed lines indicate volumetric power inputs from the power input correlation (Equation 6), corrected by the factor determined with Equation 26 (explained in context of Figure 4). Simulated conditions: Shaking diameter (*d*_0_) = 25 mm, shaking frequency (*n*) = 180–380 rpm, surface tension (*σ*) = 70 mN/m, contact angle (*θ*) = 20°, temperature (*T*) = 20°C.

After confirming that the liquid distribution is correctly simulated in the CFD at high viscosity, the model was used to calculate the volumetric power input at a variety of shaking conditions. Conditions were chosen according to already existing, experimental volumetric power input data from Büchs et al. (2000b, 2000a). Figure 2 compares the volumetric power input, computed from the CFD model and values derived from volumetric power input correlation (see Equation 6) and experiment by Büchs et al. (2000b, 2000a) at a variety of shaking conditions and viscosities. Figure 2 also shows the phase number (see Equation 4) for the given shaking conditions. Unfortunately, Büchs et al. (2000b, 2000a) do not report an experimental error for the determined volumetric power inputs. However, Büchs et al. (2000a) did include a repetition measurement after 3 months at a filling volume of 10 mL and shaking diameter of 25 mm. The repeated measurements show a maximal deviation of 0.23 kw/m^3^ and an average deviation of 0.06 kW/m^3^.

Figure 2A shows the volumetric power inputs for 37 mPa·s at 25 mL filling volume and Figure 2B,C for 59 and 97 mPa·s at 40 mL filling volume, respectively. For all three viscosities (Figure 2A–C) the volumetric power input from experiments and CFD differ remarkably little over the analyzed shaking frequencies. The average deviation between CFD and experiment is merely 0.14 kW/m^3^ or 2.3%. This indicates an exceptional performance of the CFD model, even at these near out‐of‐phase to out‐of‐phase operating conditions. Phase numbers for the investigated conditions are in the range of 1–1.5, hence already crossing the critical phase number of 1.26 by Büchs et al. (2000b). Figure 2 also depicts the volumetric power input calculated with the volumetric power input correlation by Büchs et al. (2000b) (see Equation 6). Although these values generally follow the same trajectory as the experimental data and volumetric power inputs calculated with the CFD model, they are consistently higher. In Figure 2A,B the volumetric power input correlation overestimates the volumetric power input by roughly 30%, but in Figure 2C the overestimation is on average at approximately 80%. It must be emphasized, that the volumetric power input correlation by Büchs et al. (2000b) was solely developed for fully in‐phase operating conditions (phase number larger than 1.26). In Figure 2A the phase number crosses this critical phase number of 1.26 at a shaking frequency of 300 rpm and in Figure 2B at 260 rpm In Figure 2C the phase numbers at all shaking frequencies are below this critical phase number. It is, hence, unsurprising that the overestimation by the volumetric power input correlation is larger in Figure 2C, than in A and B. It should also be mentioned, that the transition from in‐phase to out‐of‐phase is inherently a gradual transition. Hence, any one critical phase number is, although practically useful, never fully accurate. It is, therefore, unsurprising, that the volumetric power input correlation overestimates the volumetric power input at near out‐of‐phase conditions, even when the phase number is above 1.26. This again highlights the benefit of a well validated CFD model for shake flasks.

**Figure 3:**
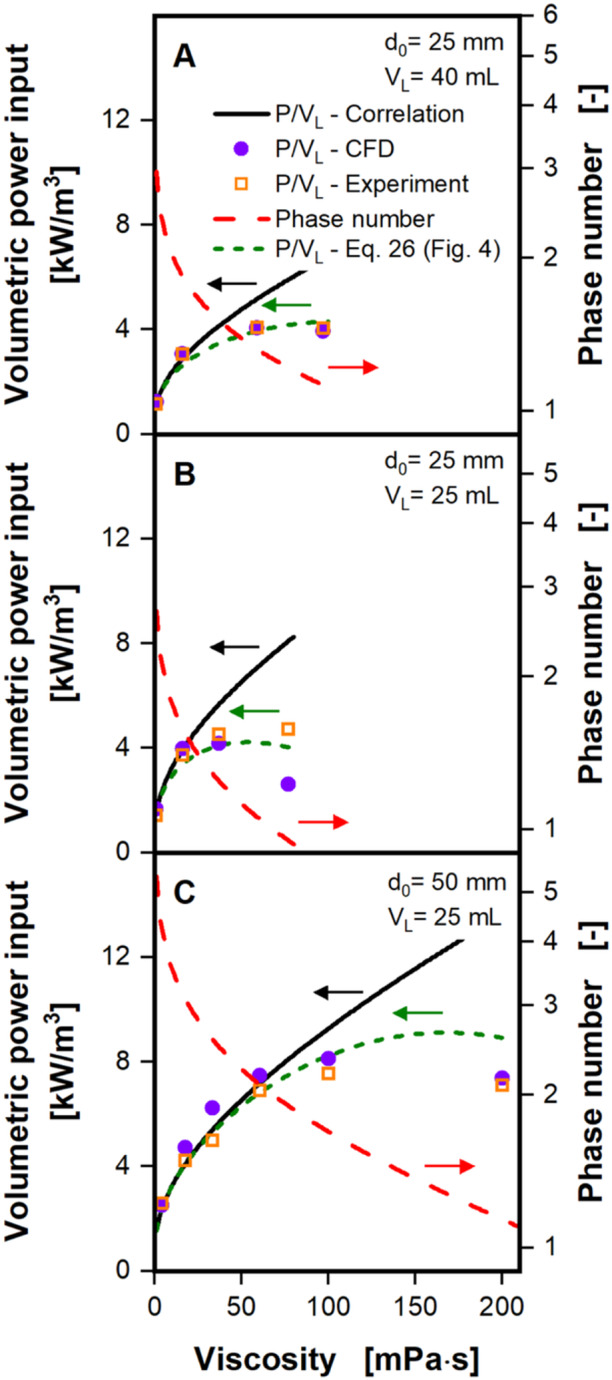
Volumetric power input and phase number as a function of viscosity. Volumetric power inputs are derived from the CFD model and compared to experimentally determined values and values calculated with the power input correlation (Equation 6), developed for in‐phase operating conditions by Büchs et al. (2000b, 2000a). Phase numbers are calculated according to Büchs et al. (2000b) (see Equation 4) and are depicted on a logarithmic scale. The green dashed lines indicate volumetric power inputs from the power input correlation (Equation 6), corrected by the factor determined with Equation (26) (explained in context of Figure 4). Simulated conditions: Shaking diameter (*d*_0_) = 25 mm (A + B) and 50 mm (C), filling volume (*V_L_*) = 40 mL (A) and 25 mL (B + C), shaking frequency ( 300 1/min, surface tension (*σ*) = 70 mN/m, contact angle (*θ*) = 20°, temperature (*T*) = 20°C, viscosity (*η*) = 1–97 (A), 1–76.8 (B), 4–199 mPa·s (C).

Figure 3 illustrates the volumetric power input over the viscosity, complementing the plot over the shaking frequency in Figure 2. Varying the viscosity allows for a larger response of the phase number and, hence, enables a clearer investigation of the performance of the CFD model from in‐phase into out‐of‐phase operating conditions. Furthermore, the limits of the volumetric power input correlation can be explored in more detail. Figure 3 depicts the volumetric power input from CFD, experiment and from the power input correlation (see Equation 6) and the phase number (see Equation 4) at a shaking diameter of 25 mm and filling volume of 40 mL (Figure 3A), 25 mm, and 25 mL (Figure 3B) and 50 mm and 25 mL (Figure 3C), all at a shaking frequency of 300 rpm and at viscosities ranging from roughly 1 mPa·s up to 200 mPa·s. Volumetric power inputs from experiments and from the CFD model, again, align exceptionally well across almost all investigated experimental conditions. In Figure 3A the deviation between experiment and CFD is on average just 0.05 kW/m^3^ (equates to 2.6%) a remarkably small difference. Figure 3B shows a similar performance for the first three viscosities of up to 37 mPa·s, with an average deviation of less than 0.3 kW/m^3^ (less than 10%). At the highest viscosity of 76.8 mPa·s in Figure 3B, the largest deviation between experimental volumetric power inputs and those from CFD can be observed. The experimental value is more than 2 kW/m^3^ (more than 80%) higher, than the CFD indicates. Possible explanations will be discussed below. Volumetric power inputs from experiment and CFD differ again very little in Figure 3C, by approximately 0.55 kW/m^3^ (less than 9%) for all viscosities. It is remarkable, that these small deviations between experiment and CFD hold across a wide range of phase numbers, from above 3 to below 1, notably crossing the critical phase number of 1.26 by Büchs et al. (2000b).

At high phase numbers, above a value of roughly 2, that is, far above both critical phase numbers and, hence, at fully in‐phase operating conditions, the volumetric power input correlation (see Equation 6) perfectly matches the volumetric power inputs from experiment and CFD. While the correlation continues to increase for smaller phase numbers, volumetric power inputs from CFD and experiment remain constant, or even decline slightly at high phase numbers. As discussed previously, the volumetric power input correlation was only developed for fully in‐phase operating conditions and, hence, cannot accurately predict the volumetric power input at out‐of‐phase conditions. The volumetric power input correlation only captures the aspect of increasing friction with increasing viscosity. The deviation between the volumetric power input correlation and CFD and experiment seems to arise roughly at phase numbers of 1.5, slightly above the critical phase number of 1.26 established by Büchs et al. (2000b). However, as discussed in context of Figure 2, a deviation between volumetric power input correlation and experiment and CFD is to be expected, even above the critical phase number, because of the inherently gradual transition from in‐phase to out‐of‐phase conditions. Interestingly, the largest deviation in volumetric power input between experiment and CFD of above 2 kW/m^3^, shown in Figure 3B, occurred at the lowest phase number in Figure 3, of roughly 0.95. Notably, this phase number is below the critical phase number of 1.26 by Büchs et al. (2000b) and is even close to the more recent critical phase number of 0.91 by Azizan et al. (2019). At phase numbers this low, it is known, that the volumetric power input tends to become unstable and even small differences in operating conditions can strongly affect the volumetric power input (Büchs et al. 2000b, 2000a, 2001). Büchs, Lotter and Milbradt (2001) showed, that for large shake flasks, even the acceleration profile to reach the desired shaking frequency might determine, whether the liquid will remain in‐phase or will be out‐of‐phase. Hence, even small differences between experimental setup and CFD might lead to the rather large differences in volumetric power input observed between experiment and CFD. Across all conditions in Figure 3, the difference in volumetric power input between experiment and CFD is only 12.4% (7.1% when disregarding the largest deviation), emphasizing the high accuracy of the CFD model and experimental volumetric power inputs.

**Figure 4:**
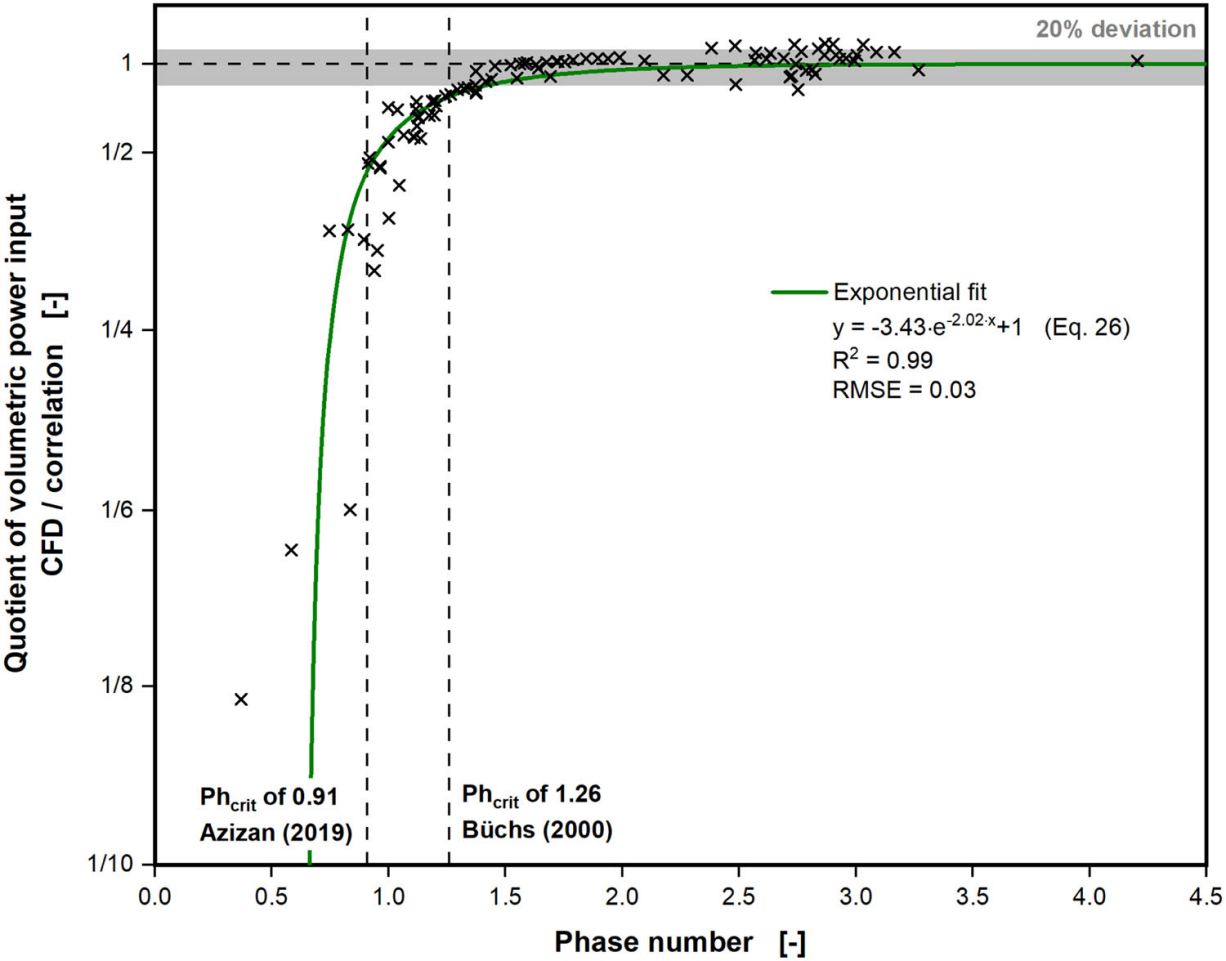
Quotient of volumetric power inputs derived from the CFD simulations and calculated with the power input correlation (see Equation 6), developed for in‐phase operating conditions by Büchs et al. (2000b), as a function of the phase number (see Eq. 25). Dashed vertical lines show the critical phase numbers for out‐of‐phase conditions of 1.26, according to Büchs et al. (2000b) and 0.91, according to Azizan et al. (2019). The horizontal gray area indicates a 20% deviation of the volumetric power inputs, derived from the CFD simulations from the volumetric power inputs, calculated with the power input correlation for in‐phase operating conditions (Büchs et al. 2000b). The green curve indicates the fitted, exponential relationship of the quotient of volumetric power input from CFD model and power input correlation with the phase number. Simulated conditions: Viscosity (*η*) = 0.69–100 mPa·s, shaking diameter (*d*_0_) = 25 and 50 mm, shaking frequency (*n*) = 150–450 rpm, filling volume (*V_L_*) = 15–40 mL, surface tension (*σ*) = 70 mN/m, contact angle (*θ*) = 20°, temperature (*T*) = 20°C–37°C.

For a more thorough estimation of the relationship of phase number and volumetric power input, the quotient of the volumetric power input from CFD and volumetric power input correlation was plotted over the phase number in Figure 4. Figure 4 contains more than 100 simulations, including simulations from our earlier work (Dinter et al. 2024). Indicated by dashed vertical lines are the two critical phase numbers, which have previously been published by Büchs et al. (2000b) and Azizan et al. (2019). Büchs et al. (2000b) derived their critical phase number from experimentally determined volumetric power inputs and Azizan et al. (2019) from experimentally determined liquid distributions. At high phase numbers, the quotient between CFD and correlation is scattered with a 20% deviation around 1. As already discussed, however, the correlation overestimates the volumetric power input, compared to the CFD, for small phase numbers. Below the critical phase number of 0.91 by Azizan et al. (2019) (left vertical dashed line) this overestimation escalates. Around phase numbers of 0.5 the CFD predicts up to eight times lower volumetric power inputs. Even at the more conservative critical phase number of 1.26 by Büchs et al. (2000b) (right vertical dashed line), the volumetric power inputs by the CFD are already consistently 30% smaller than the correlation suggests. As can be seen in Figure 4, the data points gradually approach unity with increasing phase number, without any drastic or abrupt changes, which would indicate a sudden onset of the out‐of‐phase phenomenon. Both previously published critical phase numbers (Azizan et al. 2019; Büchs et al. 2000b) are useful as a general guideline for selection of operating conditions. They, however, fail to truly capture the gradual change in fluid flow with sinking phase numbers.

Figure 4 also depicts an exponential fit (green line) of the quotient of volumetric power input from CFD and volumetric power input correlation. The fit takes the following form:

(26) $$y=-3.43\;\cdot \;e^{-2.02\cdot x}+1,$$

where *y* is the quotient of volumetric power input from CFD and correlation and *x* the phase number. This quotient enables the correction of the volumetric power input, as determined by the volumetric power input correlation at low phase numbers. In Figures 2 and 3 the quotient was already used to correct the volumetric power input of the correlation from (Büchs et al. 2000b), as indicated by the dashed green lines. In Figure 2 the corrected volumetric power input correlation (dashed green lines) is now neatly aligned with volumetric power inputs from CFD and experiments for all three viscosities and the entire range of the shaking frequency. In Figure 3 a similar performance can be observed. Only for the highest viscosities, therefore lowest phase numbers, in Figure 3B,C can a deviation between corrected volumetric power correlation (dashed green line) and values from CFD and experiment still be seen. It is, however, noteworthy, that the corrected volumetric power input correlation now displays stagnant volumetric power inputs and even declining volumetric power inputs for low phase numbers. This behavior has not been captured in the original volumetric power input correlation by Büchs et al. (2000b).

Despite the general recommendation to conduct shake flask experiments under in‐phase operating conditions, this might not always be possible, particularly at high viscosity. Peter et al. (2004) investigated the influence of the out‐of‐phase phenomenon on two previously performed screening projects of filamentous fungi. They found that initial wild type strains reached out‐of‐phase operating conditions during the fermentation, due to the filamentous growth and, hence, increasing viscosity. It turned out, that later strain generations, selected during the screening campaign, showed a successively lower maximal viscosity, remaining in‐phase during cultivation. A less filamentous morphology caused the lower maximal viscosity of later strain generations. Peter et al. (2004) underline that later strain generations were chosen, during the screening projects, based on the production titer, but that the increases in titer were simply caused by the altered morphology. The altered morphology led to in‐phase operating conditions, which improved mixing and mass transfer. Although the selection of strains with more disperse growth was unintentional in the screening projects discussed by Peter et al. (2004), deliberately exploiting the out‐of‐phase phenomenon to select for, or to manipulate fungal morphology seems promising. The presented CFD model and to some extent the correction of the volumetric power input (exponential fit, i.e., green line, in Figure 4) enable the determination of volumetric power input at the required operating conditions necessary for such a carefully planned screening campaign.

**Figure 5:**
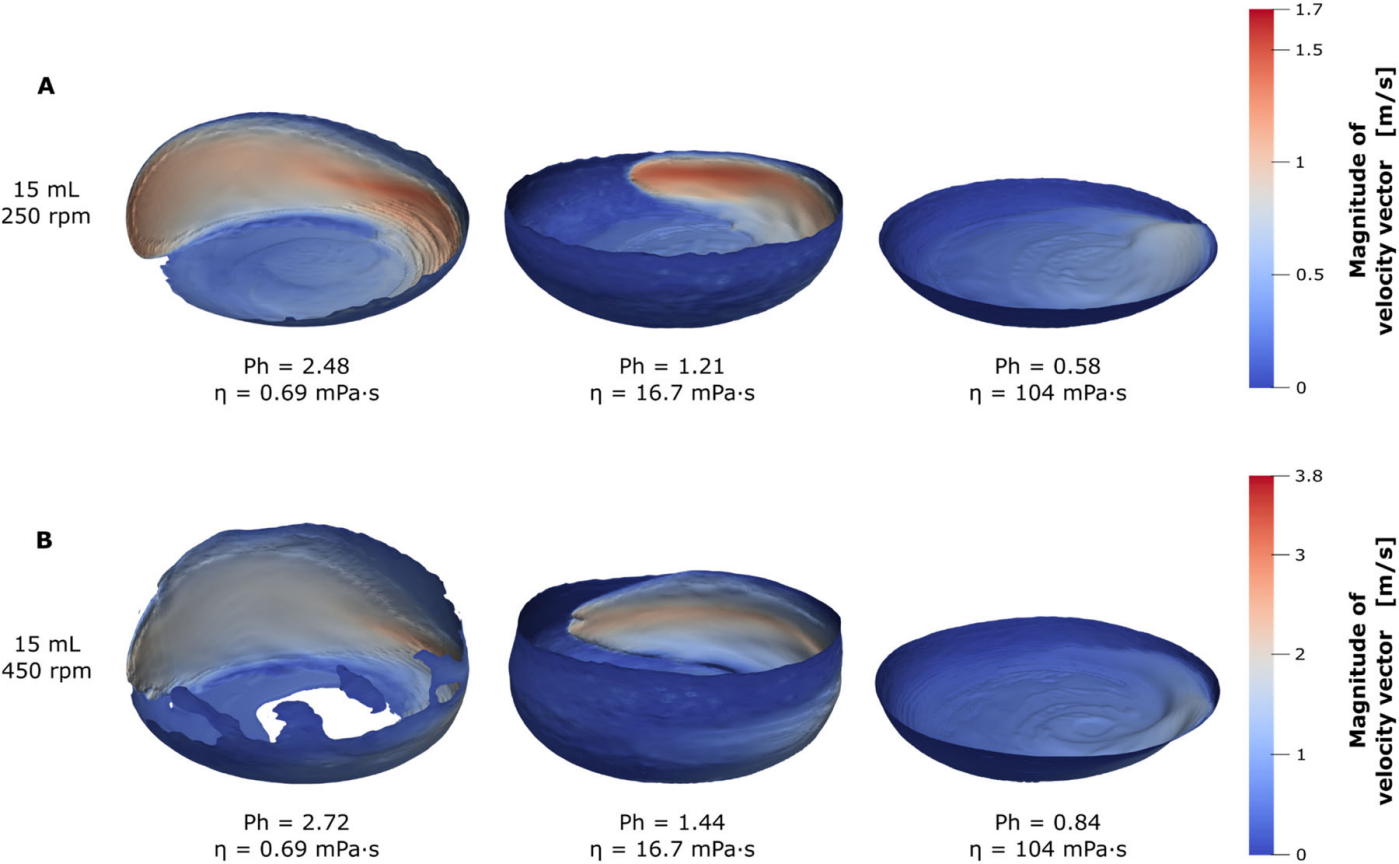
3D images of the free surface area from CFD simulations with decreasing phase numbers (see Equation 25), passing the critical phase numbers. The color scale from blue to red illustrates the magnitude of the velocity vector, easily highlighting the rotating bulk liquid in the simulations. Conditions were deliberately chosen to cross the critical phase numbers from Büchs et al. (2000b) (*Ph* = 1.26) and Azizan et al. (2019) (*Ph* = 0.91) with increasing viscosity. Simulated conditions: Viscosity (*η*) = 0.69, 16.7, and 104 mPa·s, shaking diameter (*d*_0_) = 25 mm, filling volume (*V_L_*) = 15 mL, surface tension (*σ*) = 70 mN/m, contact angle (*θ*) = 20°, temperature (*T*) = 20°C, shaking frequency (*n*) = 250 rpm (A) and 450 rpm (Β).

The deviations between volumetric power input correlation and volumetric power input from experiments and CFD, observed with decreasing phase number in Figures 2 and 3, must be connected to distinct changes in the liquid distribution. Changes in the liquid distribution are the only possible reason, why the volumetric power input would not continue to rise with increasing viscosity (see Figure 3), as is predicted by the volumetric power input correlation. While Büchs et al. (2000b) used the volumetric power input to determine their critical phase number for out‐of‐phase conditions, Azizan et al. (2019) already used their observations of the liquid distribution to determine a critical phase number. Figure 5 depicts 3D representations of the liquid distribution of a selection of CFD simulations. The color scale from blue to red corresponds to the magnitude of the velocity vector in the simulation. While Figure 5A shows the results at 15 mL filling volume with 250 rpm shaking frequency, Figure 5B shows the same filling volume at 450 rpm. For both shaking frequencies the same three viscosities were deliberately chosen to cross both critical phase numbers of 0.91 and 1.26. For the lowest viscosity of 0.69 mPa·s, corresponding to phase numbers larger than 2, the bulk liquid clearly flows along the flask wall, with the leading edge showing a gentle, rounded shape. In contrast, at the highest viscosity of 104 mPa·s, that is, phase numbers below the critical phase number of 0.91 by Azizan et al. (2019), the liquid no longer flows along the flask wall and remains instead at the bottom of the shake flask. Only a tiny wave, flowing at the bottom with greatly reduced maximal speeds can be seen in these cases. An indication of this shift in liquid distribution was already visible in Figure 1. In Figure 1G–I, the phase numbers dropped below the critical phase number of 0.91. Compared to Figure 1A–F, the maximal liquid height is greatly reduced, implying that the liquid no longer flows along the flask wall. Additionally, the shape of the observed rotating bulk liquid changed between Figure 1A–F and 1G–I, aligning with the findings from Figure 5. The intermediate viscosity of 16.7 mPa·s in Figure 5 results in phase number of 1.21 and 1.44, that is, close to the critical phase number by Büchs et al. (2000b). At these phase numbers, the liquid still flows largely along the flask wall, but the rotating bulk liquid appears more compacted, with the leading edge having a much narrower tip. Incidentally, this compacting of the rotating bulk liquid becomes more severe with decreasing phase numbers. The described change in liquid shape can be observed across both shaking frequencies for the cases above the critical phase number of 0.91 (B at 0.69 mPa·s → A at 0.69 mPa·s → B at 16.7 mPa·s → A at 16.7 mPa·s).

The findings for the volumetric power input and investigation of the liquid distribution confirm the critical phase number of 0.91 by Azizan et al. (2019). Below this phase number a complete collapse of the liquid distribution, with drastic decreases in the volumetric power input, can be observed. But they also give validity to the original critical phase number of 1.26 by Büchs et al. (2000b). Around this phase number first changes in the liquid distribution are visible, with noticeable effects on the volumetric power input. Although, the characterization in in‐phase and out‐of‐phase operating conditions remains useful in practice the transition between these states is gradual in nature, as underlined by the presented results.

### 3.3 Volumetric Gas‐Liquid Mass Transfer Rates

**Figure 6:**
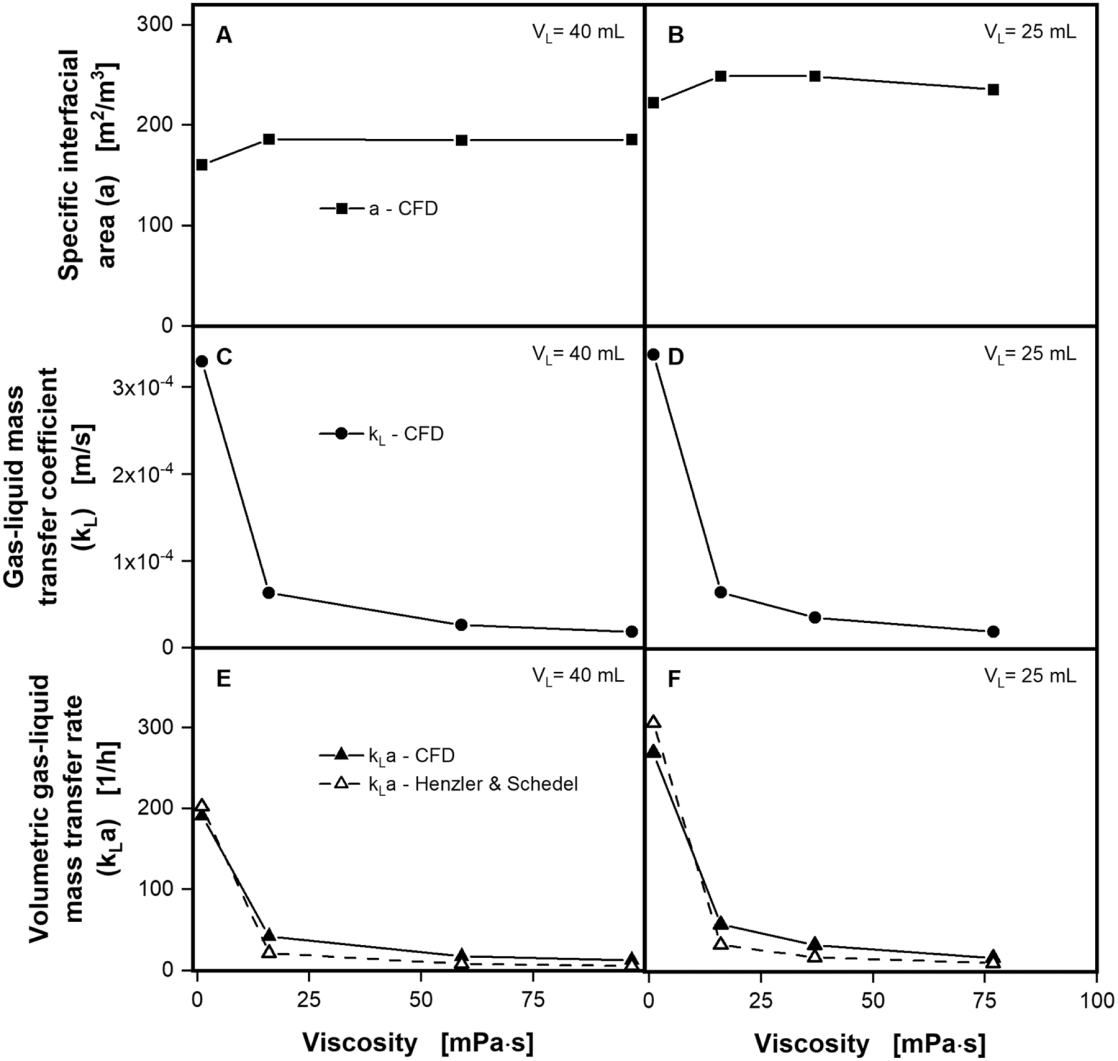
The k_L_a value derived from CFD simulations as a function of viscosity. The specific interfacial area *a* (A + B), gas–liquid mass transfer coefficient k_L_ (C + D) and volumetric gas‐liquid mass transfer rate k_L_a (E + F) are derived from the CFD simulations and depicted as a function of viscosity. The k_L_a values from the CFD simulations in E and F are compared to values calculated with the k_L_a correlation from Henzler and Schedel (1991) (see Equation 16). Simulated conditions: Shaking frequency (*n*) = 300 rpm, shaking diameter (*d*_0_) = 25 mm, surface tension (*σ*) = 70 mN/m, contact angle (*θ*) = 20°, temperature (*T*) = 20°C, viscosity (*η*) = 1–97 mPa·s (A, C, E) and 1–76.8 mPa·s (B, D, F), filling volume *V_L_* = 40 mL (A, C, E), and 25 mL (B, D, F).

The k_L_a value is another of the most important process parameters for shake flasks and bioprocesses in general. Hence, the k_L_a value was computed for the CFD simulations, investigating again the influence of increasing viscosity. Figure 6 depicts the specific interfacial area (*a*), the gas–liquid mass transfer coefficient (k_L_) and the k_L_a value at a filling volume of 40 mL (Figure 6A,C,E) and 25 mL (Figure 6B,D,F) at 300 rpm and viscosities from 1 mPa·s up to 97 mPa·s. A wide variety of other shaking conditions are included in the supplement in Supporting Information: Figure S3–S6.

The specific interfacial area (a) stays almost constant with increasing viscosity, only showing a small decline at the highest viscosity at 25 mL (see Figure 6B). This finding is consistent with the analysis by Henzler and Schedel (1991), who also found that the specific interfacial area (a) is not significantly affected by the viscosity. As expected, the reduction of filling volume, at otherwise constant shaking conditions, leads to a higher specific interfacial area (a) (compare Figure 6A,B). Further, specific interfacial area (a) is roughly 25 m^2^/m^3^ lower at the waterlike viscosity of 1 mPa·s, compared to the next higher viscosity at both filling volumes. This underestimation at waterlike viscosity is most likely a shortcoming of the presented CFD model. The mesh size of the CFD model is probably too coarse to fully resolve the liquid film of roughly 50 µm, expected at waterlike viscosity. The tradeoff of computational effort and model accuracy was mentioned in Dinter et al. (2024). The effect can be seen in Figure 5, where the liquid film is fully resolved for the higher viscosities, but not for the waterlike viscosity. For the two higher viscosities in Figure 5 the liquid film (dark blue area) reaches up to the maximal liquid height over the entire circumference of the shake flasks, while this is not the case for the waterlike viscosity.

The k_L_ decreases sharply with increasing viscosity at both filling volumes (Figure 6C,D), dropping from almost 3 × 10^−4^ m/s to about 0.5 × 10^−4^ m/s at 16 mPa·s and decreasing further to below 0.2 × 10^−4^ m/s at the highest respective viscosity. This decline of the k_L_ is directly caused by the reduction of the diffusion coefficient and changes in liquid distribution due to the increasing viscosity. The k_L_a value, being the product of k_L_ and a, shows the combination of both signals, again sharply decreasing with moderately increasing viscosity. Values computed with the CFD model (see Equation 15) are compared to k_L_a values calculated with the k_L_a correlation from Henzler and Schedel (1991) (see Equation 16). The progression of the k_L_a value from CFD and k_L_a correlation are exceptionally similar. At waterlike viscosity the k_L_a value from the CFD is 5% (Figure 6E) and 12% (Figure 6F) lower, than indicated by the k_L_a correlation from Henzler and Schedel (1991). For the higher viscosities, the k_L_a value from the CFD model is approximately twice as high as indicated by the k_L_a correlation from Henzler and Schedel (1991). It should be noted, that, both, the CFD method and the k_L_a correlation from Henzler and Schedel (1991) require the diffusion coefficient for the k_L_a value calculation. In this work the diffusion coefficient is calculated according to Eqquation (12), which gives the diffusion for pure oxygen in pure water, including viscous effects. It, however, does not include effects of other cultivation media components like salt on the diffusion coefficient. Fortunately, CFD method and k_L_a correlation from Henzler and Schedel (1991) include the diffusion coefficient with the exact same weighting, therefore it does not affect the comparison between the two.

**Figure 7:**
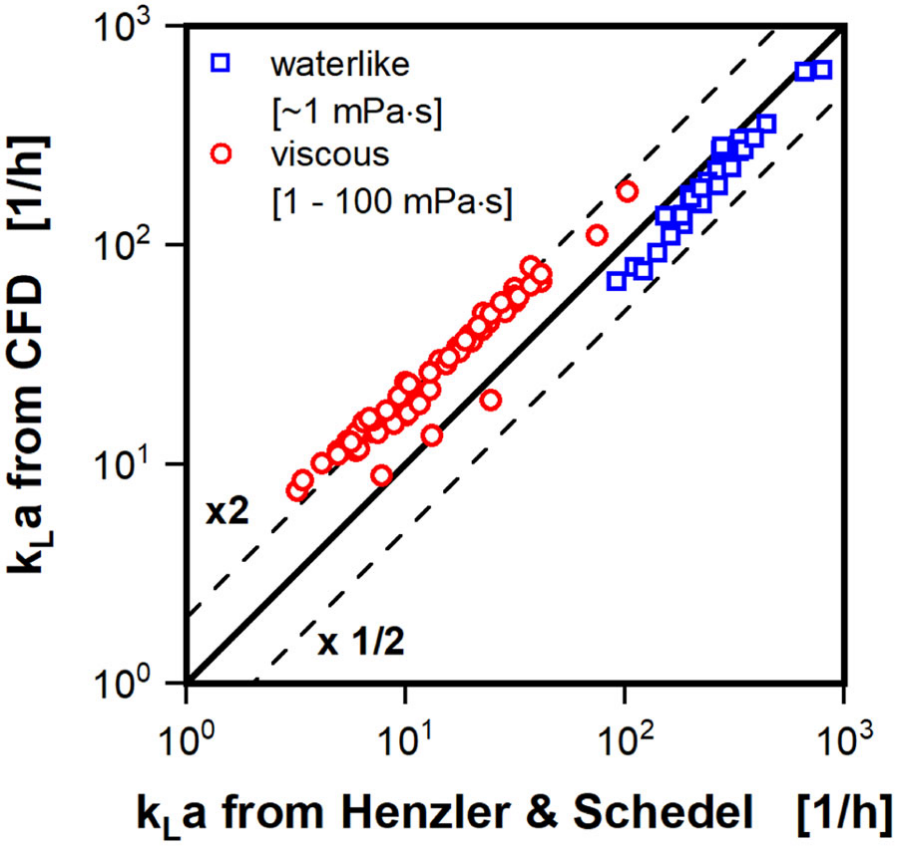
Parity plot of k_L_a values derived from the computational fluid dynamics (CFD) simulations against values calculated with the k_L_a correlation from Henzler and Schedel (1991) (see Equation 16). Simulated conditions are split into two groups; waterlike viscosity of up to 1 mPa·s (blue open squares) and elevated viscosity of above 1–100 mPa·s (red open circles). Black lines indicate parity and an over‐ and underestimation by a factor of 2. Simulated conditions: Viscosity (*η*) = 0.69–100 mPa·s, shaking diameter (*d*_0_) = 25 and 50 mm, shaking frequency (*n*) = 150–450 rpm, filling volume (*V_L_*) = 15–40 mL, surface tension (*σ*) = 70 mN/m, contact angle (*θ*) = 20°C, temperature (*T*) = 20°C–37°C.

Figure 7 shows a parity plot of both CFD and kLa correlation for a more detailed comparison, containing more than 100 simulations, including simulations from our earlier work (Dinter et al. 2024). Data points are presented separately for waterlike conditions of around 1 mPa·s (open blue squares) and for the more viscous conditions above 1 mPa·s (open red circles). The k_L_a values in Figure 7 span three orders of magnitude, from approximately 5 h^−1^ to 750 h^−1^. Across this entire range the data points follow the parity line quite closely. Data points for the waterlike viscosity are especially close to the parity line. On average the k_L_a values from the CFD model are less than 20% smaller than suggested by the k_L_a correlation. For the higher viscosity, the difference between CFD model and k_L_a correlation is somewhat larger, but with data points remaining highly parallel to the parity line. On average the k_L_a values form the CFD model are twice as high, as indicated by the k_L_a correlation (+93% with a standard deviation of roughly 32%). As discussed previously, the liquid film is not fully resolved in the CFD model for waterlike viscosity. The liquid film has, however, previously been proven to contribute significantly to the overall oxygen transfer in shake flasks experiments (Gaden 1962; Giese et al. 2014; Maier, Losen, and Büchs 2004; Maier and Büchs 2001; Meier et al. 2016). Maier et al. showed, that the maximal oxygen transfer capacity in hydrophobic shake flasks, where no liquid film is formed, was reduced by up to a factor of four (Maier and Büchs 2001). Hence, the not fully resolved liquid film in the CFD model might explain, why the CFD model produces k_L_a values lower than the k_L_a correlation at low viscosity, but higher than the k_L_a correlation at higher viscosities. Liu et al. (2016) showed a similar deviation between k_L_a values computed by their CFD model for shake flasks and the k_L_a correlation by Henzler and Schedel (1991) for waterlike viscosity, albeit for only four CFD simulations, performed at similar shaking conditions. Interestingly, their CFD model apparently also did not fully resolve the liquid film.

The k_L_a computation in the CFD model uses the mass transfer model from Lamont and Scott (1970) (see Equation 13), which focuses on the influence of small eddies near the interface surface, as the acting force behind the mass transfer. Lamont and Scott (1970) compared the results of their mathematical approach for the calculation of the k_L_ to three widely different experimental situations. First, they compared it to their own experimental work for bubbles in a turbulent pipe flow. Second, to the experimental data from Calderbank and Moo‐Young (1961) for bubble aerated stirred tank reactors including a solid phase and thirdly, to the experimental data from Fortescue and Pearson (1967) for the transfer into the surface of turbulent channel flow. Lamont and Scott (1970) also found a deviation within a factor of 2 between their model and the three experimental data sets.

For the correlation of the k_L_a for shake flasks, Henzler and Schedel (1991) determined oxygen transfer rates for experiments with the sulfite system and culture broth of *Escherichia coli* and *Streptomyces tendae*. They used the resulting oxygen transfer rates to calculate the k_L_a value under the assumption of a complete absence of oxygen in the liquid phase (dissolved oxygen tension = 0) (Henzler and Schedel 1991). However, Meier et al. (2016) later showed, that the dissolved oxygen tension does not reach zero in the liquid film. Hence, the correlation by Henzler and Schedel likely underestimates the k_L_a value for shake flasks to some degree. Furthermore, Henzler and Schedel provide, to our knowledge, the only k_L_a correlation, for which the influence of the viscosity was fitted based on experimental results. However, there are some unfortunate limitations to the data used for elevated viscosity. Even though, Henzler and Schedel (1991) measured the shear‐thinning behavior of the utilized *Streptomyces tendae* culture broth, they were forced to assume a fixed shear rate (100 s^−1^) to estimate a viscosity for their model. The later developed correlation for effective shear rates would have allowed for a more precise estimation of the shear rate and, hence, viscosity (Giese et al. 2014). Additionally, Henzler and Schedel (1991) varied the viscosity by performing experiments with the sulfite system using water at various temperatures and glucose solutions of different concentrations. While this approach does not suffer the same problems of shear‐thinning behavior as the *Streptomyces tendae* culture broth, it unfortunately only covers viscosities from roughly 0.6 to 5 mPa·s. Hence, the k_L_a correlation by Henzler and Schedel (1991) might be more accurate at waterlike and low viscosities, than at higher viscosities. Given the discussed accuracy of the transfer model from Lamont and Scott (1970) and shortcoming of the k_L_a correlation by Henzler and Schedel (1991), the performance of the CFD model, predicting the k_L_a value across three orders of magnitude is satisfactory.

### 3.4 Shear Rates

**Figure 8:**
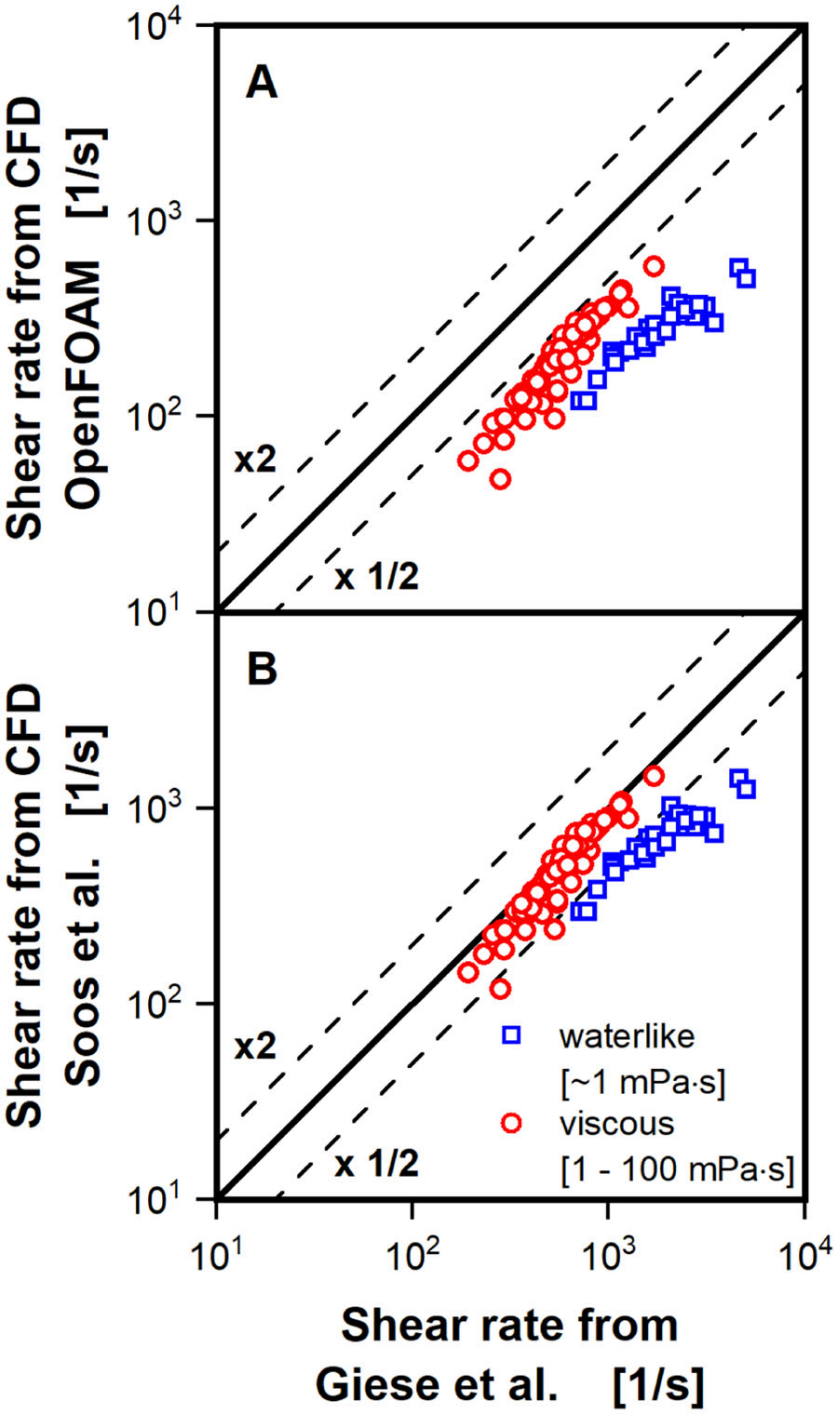
Parity plots of two approaches to derive shear rates from the CFD simulations against values calculated with the correlation from Giese et al. (2014) (see Equation 25). Simulated conditions are split into two groups; waterlike viscosity of up to 1 mPa·s (blue open squares) and elevated viscosity above 1–100 mPa·s (red open circles). Black lines indicate parity and an over‐ and underestimation by a factor of 2. In (A) the shear rate calculation is the one implemented in OpenFOAM. In (B) the shear rate calculation for the CFD model is based on the calculation of shear stress provided by Soos et al. (2010). Simulated conditions: Viscosity (*η*) = 0.69–100 mPa·s, shaking diameter (*d*_0_) = 25 and 50 mm, shaking frequency (*n*) = 150–450 rpm, filling volume (*V_L_*) = 15–40 mL, surface tension (*σ*) = 70 mN/m, contact angle (*θ*) = 20°, temperature (*T*) = 20°C–37°C.

In this work, viscosity has proven to have a major influence on liquid distribution, volumetric power input and k_L_a value. However, as stated previously, the usually encountered shear‐thinning behavior of most biopolymers and filamentous fungi was approximated with a constant viscosity for Newtonian flow, based on the correlation for effective shear rates by Giese et al. (2014). Ideally, the CFD model would directly incorporate the non‐Newtonian behavior by calculating shear rates in each mesh cell. OpenFOAM, in fact, provides a scheme to calculate the shear rate (Equation 19). A set of CFD simulations, modeling the shear‐thinning behavior with the power law, has been performed for this work. Simulations were performed with the same conditions as shown in Figure 1 (except for the viscosity). Resulting liquid contact lines are shown in Supporting Information: Figure S7 and can be compared with Figure 1. Due to the very weak shear‐thinning behavior of the polyvinylpyrrolidone (flow behavior index of 0.956) used by Azizan et al. (2017, 2019), the effect on the computed liquid contact lines, of directly modeling the non‐Newtonian behavior is almost non‐existent. For a better insight into the performance of the OpenFOAM scheme for the shear rate calculation, resulting shear rates of all performed simulations are compared to the effective shear rates calculated with the correlation from Giese et al. (2014) in the parity plot in Figure 8A.

All data points are again split into waterlike and higher viscosity. It should be noted that the determination of a shear rate, in context of shear‐thinning behavior, is only truly relevant for elevated viscosities above waterlike viscosity. At waterlike viscosity (blue open squares) the shear rates calculated with the OpenFOAM scheme are on average roughly six times smaller than suggested by the correlation for effective shear rates. At the higher viscosity the performance is much better, showing only three times smaller values. As the overall performance of the OpenFOAM scheme, compared to the correlation for effective shear rates was not satisfactory, an additional approach for computing the shear rates was investigated. This approach is based on the computation of average shear stress, as given by Soos et al. (2010) (Equation 20). According to Soos et al. (2010) this calculation of average shear stress is valid only for laminar flow conditions. Although, transitional flow is to be expected for waterlike viscosity, liquid flow is laminar at higher viscosities in shake flasks. Importantly, the estimation of shear rates is mostly of interest for higher viscosities, where shear‐thinning behavior might need to be modeled. Under the assumption of Newtonian flow, the computed shear stress can be easily transformed to the shear rate. A resulting parity plot, comparing the results to the correlation for effective shear rates by Giese et al. (2014) is shown in Figure 8B. Generally, the data points are much closer to the parity line than for the OpenFOAM scheme. For waterlike viscosity, the correlation for effective shear rates now only predicts values to be 2.6 times smaller than the CFD model indicates. For the higher viscosity this difference is reduced to just 16%. For both methodologies for the calculation of the shear rate in the CFD model, the larger underestimation for the waterlike viscosity is surprising. The only apparent difference to the simulations at the higher viscosity is the not fully resolved liquid film. However, as fully resolving the liquid film was not necessary to correctly predict the volumetric power input in this work, as well as in our previous work (Dinter et al. 2024), the importance of the film was not expected. Again, estimation of the shear rate, with the aim of simulating shear‐thinning behavior, is more relevant for elevated viscosities. Although the second presented approach appears to model the shear rate much more accurately, especially for elevated viscosities, further research into its validity is necessary. Notably, the conversion of shear stress to shear rate is no longer straight forward, when non‐Newtonian conditions are simulated in the CFD. An iterative approach of estimating the shear rate with an approximation of the viscosity in the mesh cell, calculation of the shear rate and following adjustment of the viscosity in the mesh cell would be necessary.

## 4 Conclusions

The presented CFD model for 250 mL shake flasks predicts fluid flow, volumetric power input, k_L_a and shear rates for a wide variety of shaking conditions, ranging from fully in‐phase operating conditions at low viscosity to fully out‐of‐phase operating conditions at high viscosity of up to 100 mPa·s. The inherently gradual nature of the transition from in‐phase to out‐of‐phase operating conditions is highlighted by the results from the CFD model. Although defining a critical phase number to differentiate between in‐phase and out‐of‐phase conditions, as done by Büchs et al. (2000b) and Azizan et al. (2019), might be useful for a rough classification for routine lab applications, any one, single number fails to capture the complexity of fluid flow.

## Nomenclature

CFD: Computational Fluid Dynamics
d: largest inner flask diameter, mm or m
*d_0_*
: shaking diameter, cm or m
D_L_
: diffusion coefficient, m^2^/s
*f*_*GW*_
: Gierer and Wiertz correction factor, —
F_σ_
: surface tension, N/m
g: gravitational acceleration, m/s^2^
K: flow consistency index, Pa·s^m^
*k_B_*
: Boltzmann constant, J/K
k_L_a: volumetric gas‐liquid mass transfer rate, h^−1^ or s^−1^
m: flow behavior index, —
*MW*
: molecular weight, Da
n: shaking frequency, min^−1^ or s^−1^
*N_A_*
: Avogadro's constant, mol^−1^
*Ne'*
: modified Newton number, —
P: power input, W or kg·m^2^/s^3^
*Ph*
: phase number, —
Re: Reynolds number, —
S: strain rate tensor, s^−1^
Re_film_
: Reynolds film number, —
*r_M_*
: molecule radius, Å
T: temperature, K
U: velocity vector, m/s
*V_L_*
: filling volume, mL or L
*α*
: volume fraction, —
*β*
: ratio of radii of solute and solvent, —
γ: shear rate, s^−1^
*ε*
: energy dissipation rate, W/kg or m^2^/s^3^
*η*
: dynamic viscosity, Pa·s
η_t_
: turbulent dynamic viscosity, Pa·s
ν: kinematic viscosity, m^2^/s
*ρ*
: density, kg/m^3^
*ρ_eff_*
: effective density, kg/m^3^
*τ_L_*
: average shear stress, Pa

## Author Contributions

Carl Dinter performed CFD simulations, analyzed and interpreted the data and drafted the manuscript. Andreas Gumprecht performed CFD simulations. Matthias Alexander Menze helped implementing the calculations of the volumetric power input. Amizon Azizan provided the experimental liquid distribution data. Jochen Büchs and Sven Hansen initiated the study, helped revise the manuscript and provided valuable insights in the design of the study. All authors read and approved the final manuscript.

## Conflicts of Interest

The authors declare no conflicts of interest.

## Supporting information

Supporting information.

## Data Availability

The data that support the findings of this study are available from the corresponding author upon reasonable request.
