# Supplementary material for: Exploration of the Out‐of‐Phase Phenomenon in Shake Flasks by CFD Calculations of Volumetric Power Input, kLa Value and Shear Rate at Elevated Viscosity
Source: Biotechnol Bioeng. 2024 Nov 30;122(3):509–24. doi: 10.1002/bit.28892 (PMC11808426; doi:10.1002/bit.28892)
Supplement: Supplementary file 1 — Supporting information. [file BIT-122-509-s001.docx]

**Supplement: Exploration of the out-of-phase phenomenon in shake flasks by CFD calculations of volumetric power input, k_L_a and shear rate at elevated viscosity**


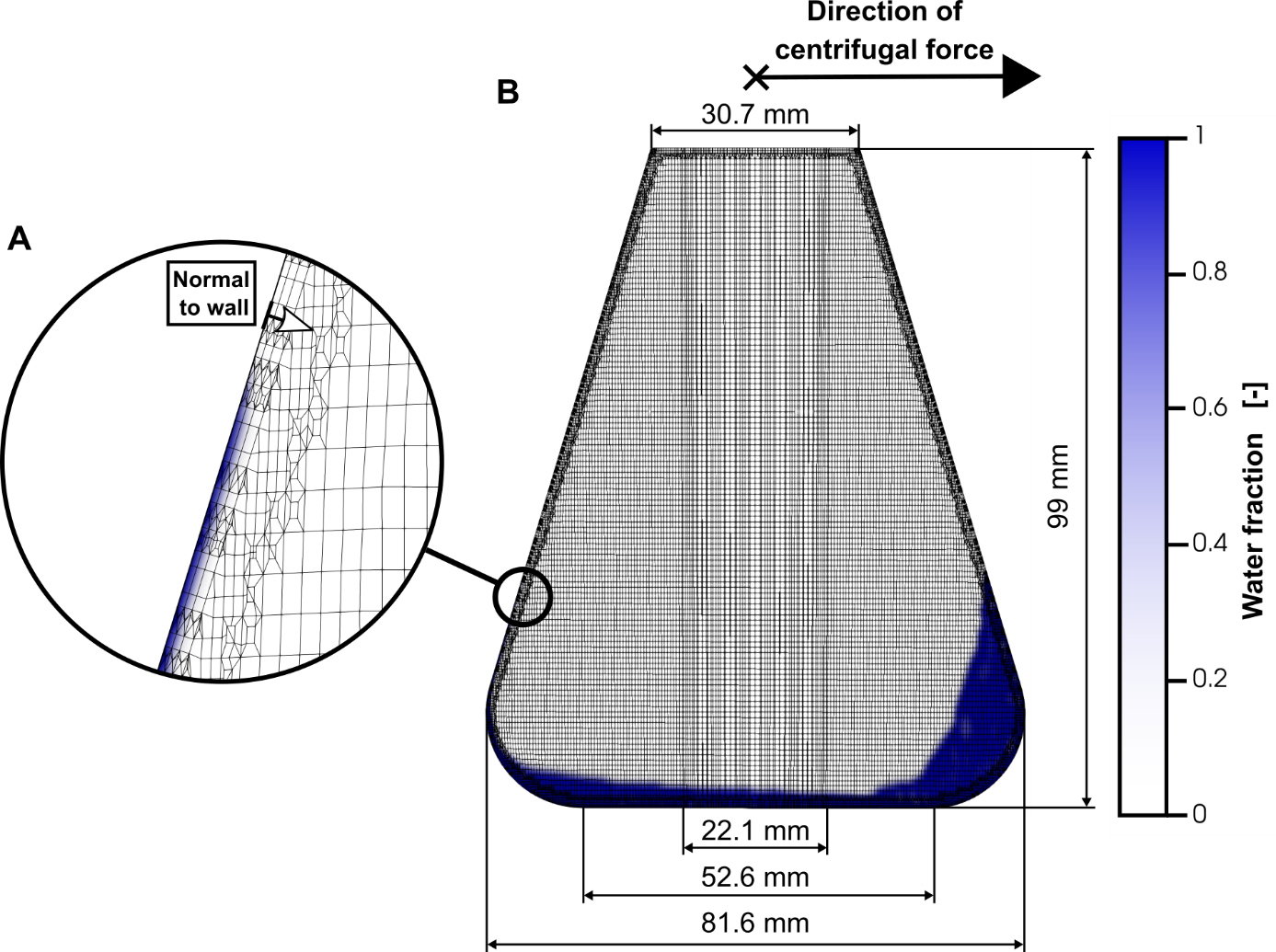


Fig. S1: Cross-section of the meshed shake flask (Image taken from Dinter et al. (2024)).

In (**B**) the cross-section of the entire shake flask mesh can be seen. A close-up view of the wall is depicted in (**A**). The 3D model of the shake flask is meshed with the OpenFOAM utility *snappyHexMesh*. First, a cylindrical base mesh is created with *blockMesh*. Two refinement steps (factor 8 increase in mesh resolution), introduced during the castellated meshing of *snappyHexMesh* can be seen. One refinement step is introduced at a diameter of 22.1 mm. A second one can be seen near the shake flask wall in (**A**). Additionally, three wall layers, depicted in (**A**) are introduced. Wall layers are orientated parallel to the shake flask wall, as indicated by the arrow normal to the wall in (**A**). A completely flat bottom with a diameter of 52.6 mm is modelled. In total, the meshed shake flask model consists of roughly 1.4 million cells. The color scale from white to blue indicates the water fraction of the simulated CFD case. Simulated conditions: Viscosity (η) = 16.7 mPa·s, shaking diameter (d_0_) = 2.5 cm, filling volume (V_L_) = 40 mL, shaking frequency (n) = 250 rpm, surface tension (σ) = 70 mN/m, contact angle (θ) = 20°, temperature (T) = 25°C

The shown mesh of the shake flasks resulted in y^+^ values from 1 to 5 for waterlike viscosity and y^+^ values below 1 for all elevated viscosities.


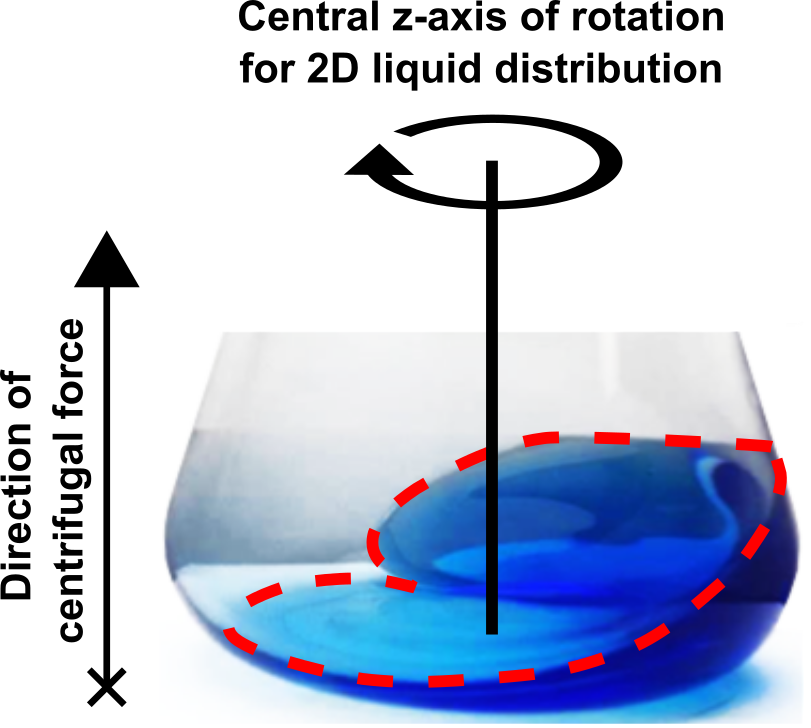


Fig. S2: Liquid distribution at a viscosity of 82.5 mPa·s (Image adapted from Dinter et al. (2024)).

Photograph of the rotating liquid in a shake flask, taken with a rotating camera, which is always pointing in the direction of centrifugal force and, hence, in a fixed position to the bulk liquid. With the dashed red line, the liquid contact line at the intersection of liquid, glass and air is highlighted. This contact line is considered representative for the entire liquid distribution and responds to changes in the operating conditions. Shaking conditions: Filling volume (V_L_) = 30 mL, shaking frequency (n) = 150 rpm, shaking diameter (d_0_) = 5 cm, temperature (T) = 25°C.


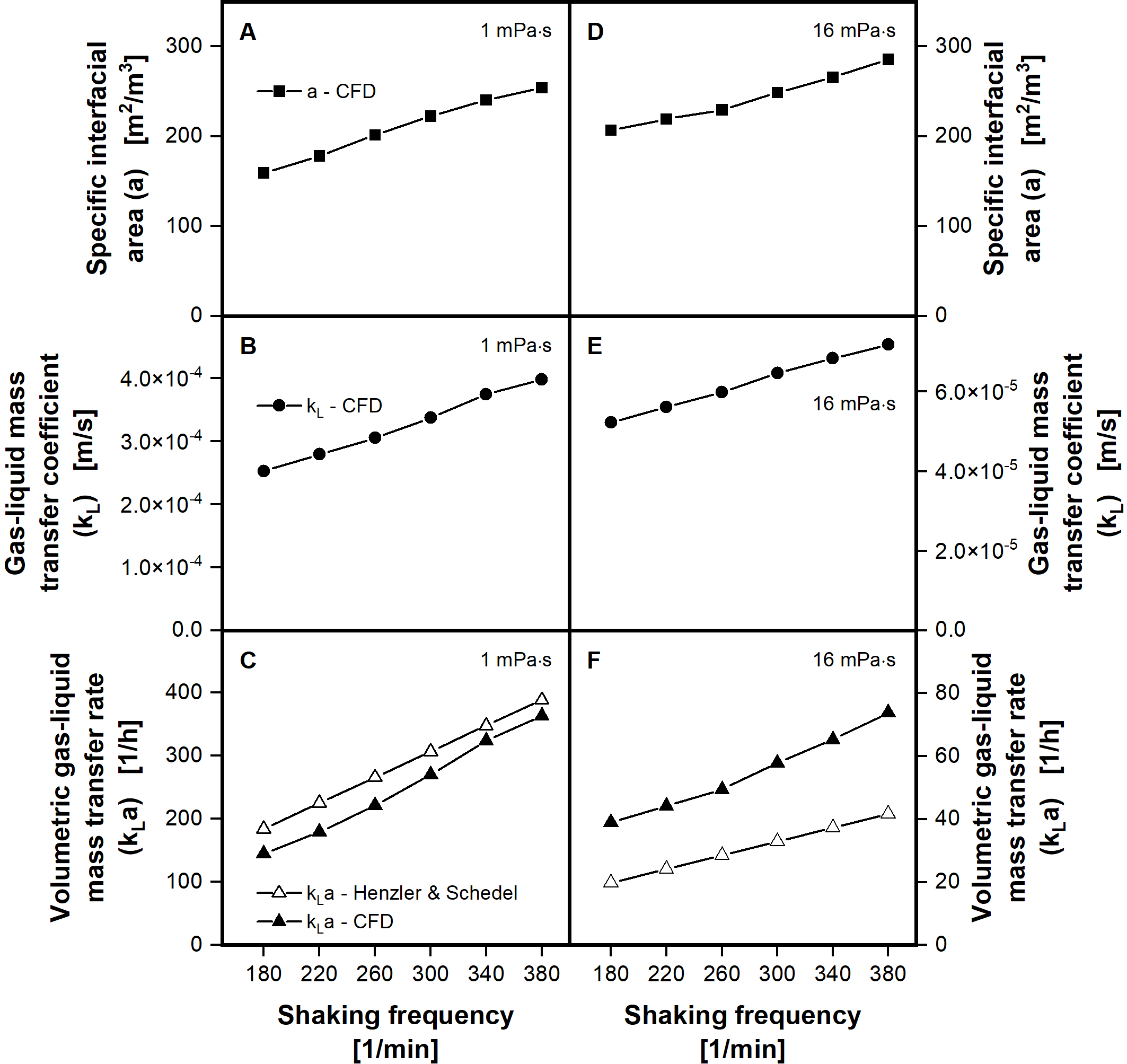


Fig. S3: The k_L_a value derived from CFD simulations as function of shaking frequency at a filling volume of 40 mL and viscosity of 1 and 16 mPa·s.

The specific interfacial area a (A + D), gas-liquid mass transfer coefficient k_L_ (B + E) and volumetric gas-liquid mass transfer rate k_L_a (C+ F) are derived from the CFD simulations and depicted as a function of viscosity. The k_L_a values from the CFD simulations in C and F are compared to values calculated with the k_L_a correlation from Henzler & Schedel (Eq. 14). Simulated conditions: Shaking frequency (n) = 180 - 340 rpm (A – C) and 180 – 380 rpm (D – F), shaking diameter (d_0_) = 25 mm, surface tension (σ) = 70 mN/m, contact angle (θ) = 20°, temperature (T) = 20 °C, viscosity (η) = 1 mPa·s (A – C) and 16 mPa·s (D – F), filling volume V_L_ = 40 mL


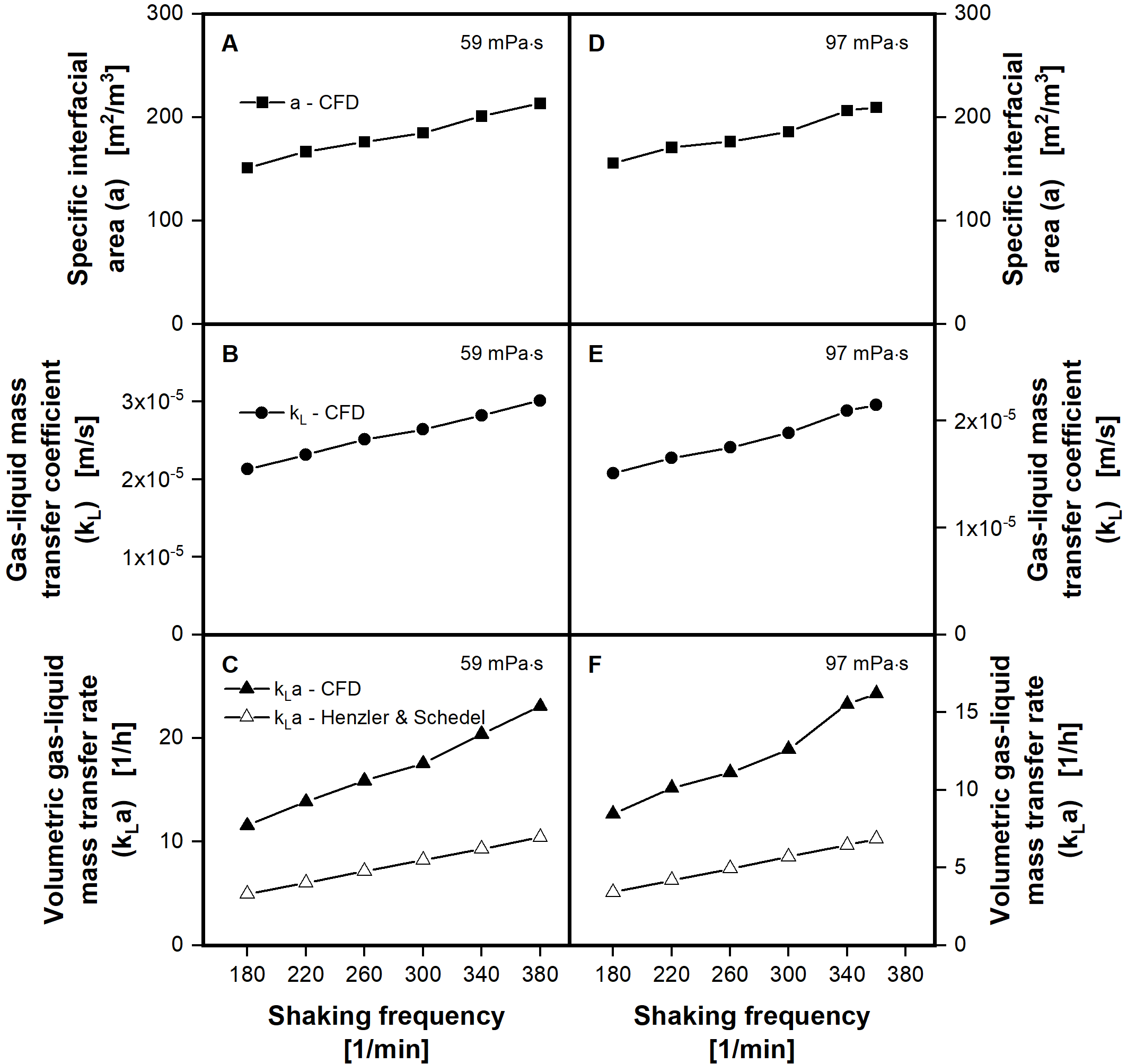


Fig. S4: The k_L_a value derived from CFD simulations as function of shaking frequency at a filling volume of 40 mL and viscosity of 59 and 97 mPa·s.

The specific interfacial area a (A + D), gas-liquid mass transfer coefficient k_L_ (B + E) and volumetric gas-liquid mass transfer rate k_L_a (C+ F) are derived from the CFD simulations and depicted as a function of viscosity. The k_L_a values from the CFD simulations in C and F are compared to values calculated with the k_L_a correlation from Henzler & Schedel (Eq. 14). Simulated conditions: Shaking frequency (n) = 180 - 348 rpm (A – C) and 180 – 360 rpm (D – F), shaking diameter (d_0_) = 25 mm, surface tension (σ) = 70 mN/m, contact angle (θ) = 20°, temperature (T) = 20 °C, viscosity (η) = 59 mPa·s (A – C) and 97 mPa·s (D – F), filling volume V_L_ = 40 mL


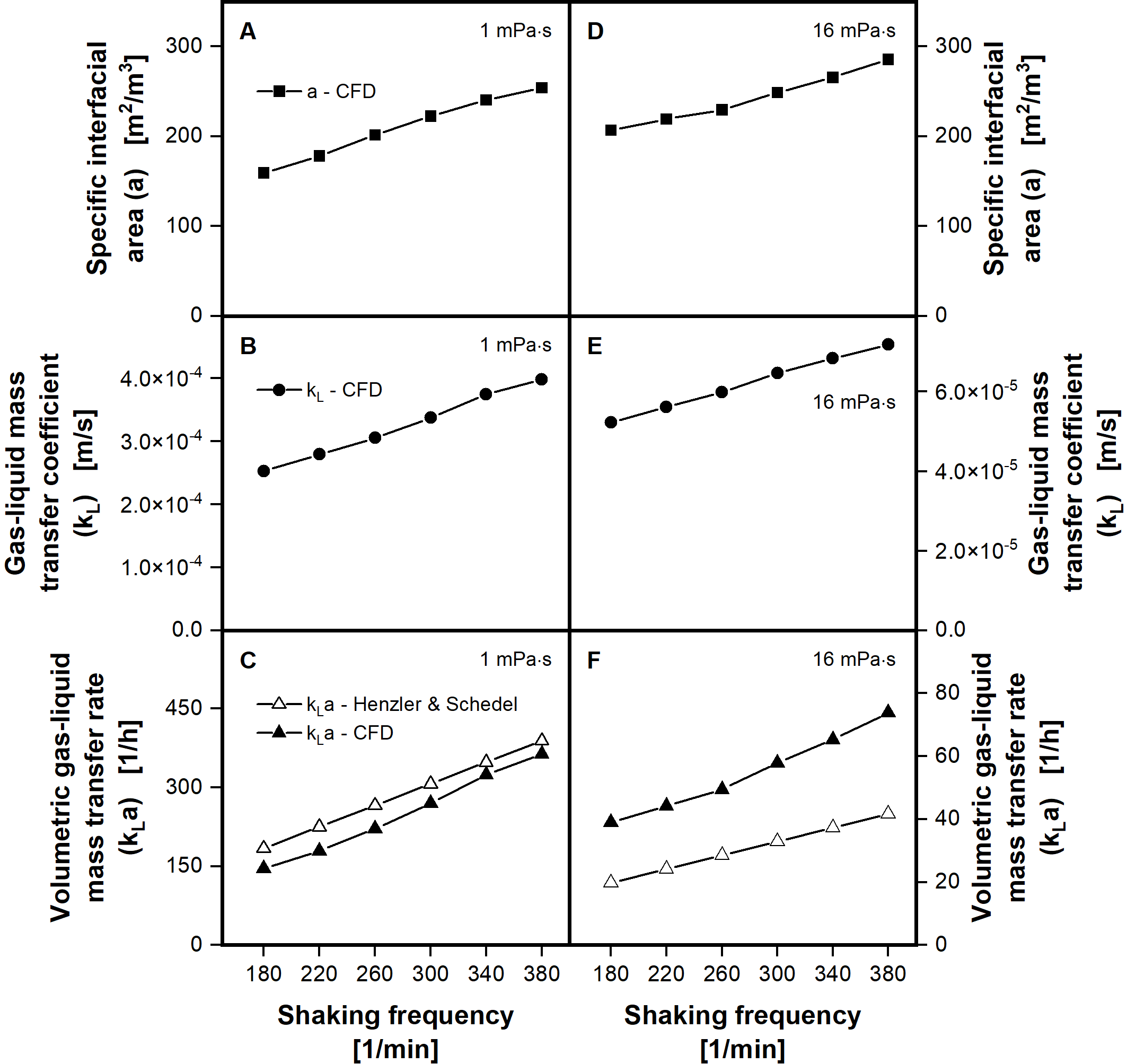


Fig. S5: The k_L_a value derived from CFD simulations as function of shaking frequency at a filling volume of 25 mL and viscosity of 1 and 16 mPa·s.

The specific interfacial area a (A + D), gas-liquid mass transfer coefficient k_L_ (B + E) and volumetric gas-liquid mass transfer rate k_L_a (C+ F) are derived from the CFD simulations and depicted as a function of viscosity. The k_L_a values from the CFD simulations in C and F are compared to values calculated with the k_L_a correlation from Henzler & Schedel (Eq. 14). Simulated conditions: Shaking frequency (n) = 180 - 380 rpm, shaking diameter (d_0_) = 25 mm, surface tension (σ) = 70 mN/m, contact angle (θ) = 20°, temperature (T) = 20 °C, viscosity (η) = 1 mPa·s (A – C) and 16 mPa·s (D – F), filling volume V_L_ = 25 mL


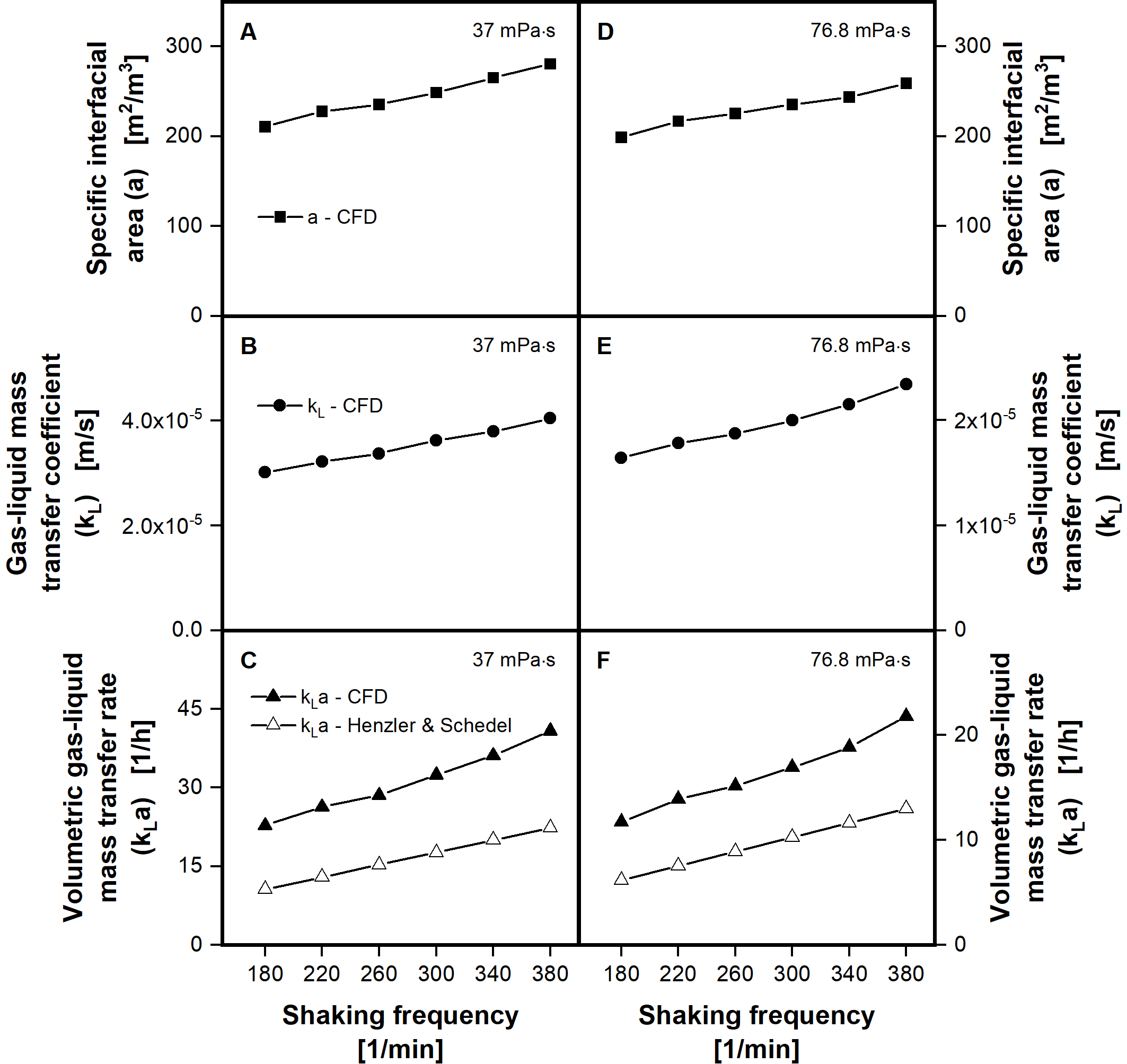


Fig. S6: The k_L_a value derived from CFD simulations as function of shaking frequency at a filling volume of 25 mL and viscosity of 37 and 76.8 mPa·s.

The specific interfacial area a (A + D), gas-liquid mass transfer coefficient k_L_ (B + E) and volumetric gas-liquid mass transfer rate k_L_a (C+ F) are derived from the CFD simulations and depicted as a function of viscosity. The k_L_a values from the CFD simulations in C and F are compared to values calculated with the k_L_a correlation from Henzler & Schedel (Eq. 14). Simulated conditions: Shaking frequency (n) = 180 - 380 rpm, shaking diameter (d_0_) = 25 mm, surface tension (σ) = 70 mN/m, contact angle (θ) = 20°, temperature (T) = 20 °C, viscosity (η) = 37 mPa·s (A – C) and 76.8 mPa·s (D – F), filling volume V_L_ = 25 mL


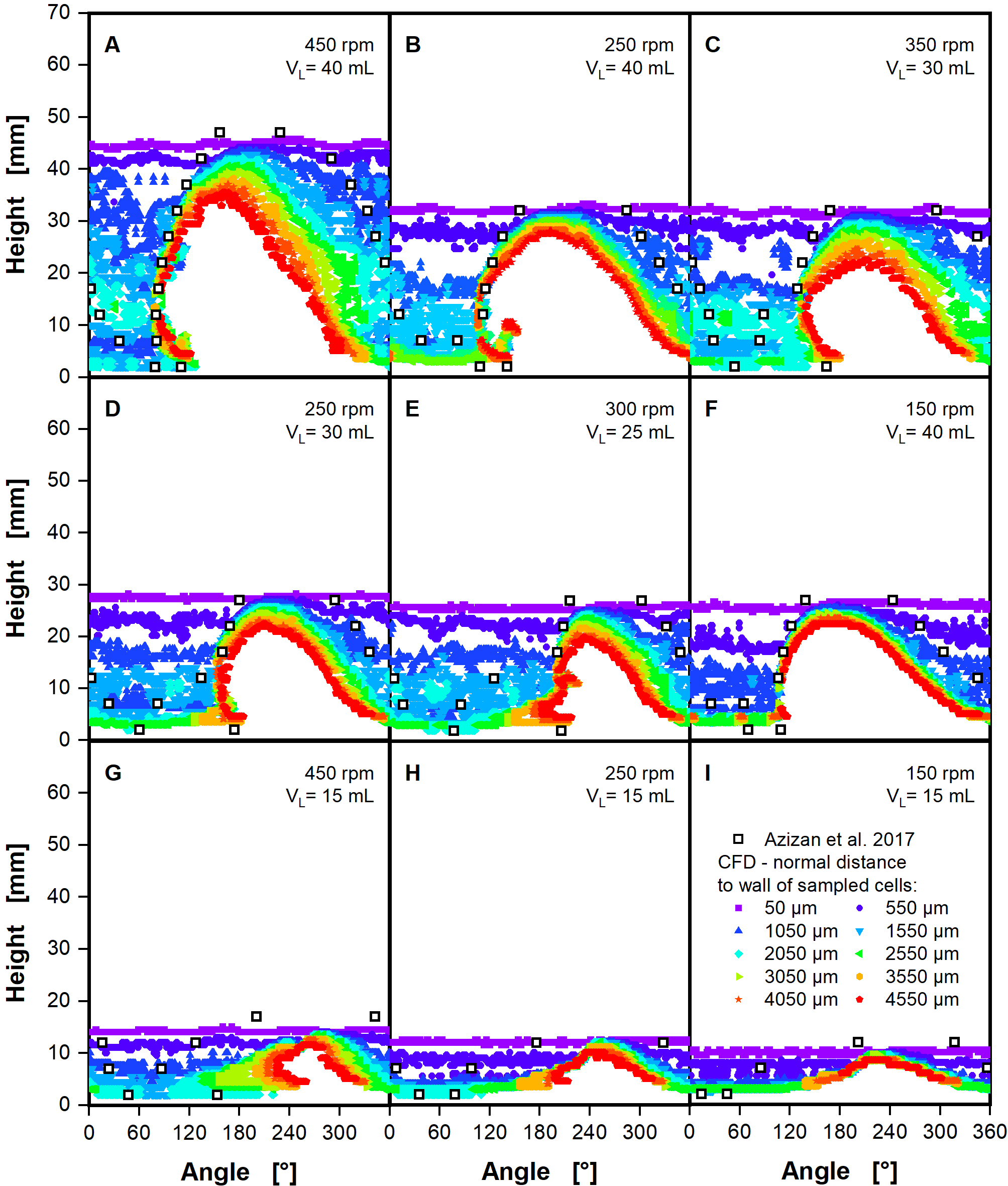


Fig. S7: Liquid contact lines extracted from the CFD model, simulating a power law, shear thinning liquid with the calculation of shear rates, as provided by OpenFOAM.

The liquid contact lines are shown in mm, viewed from the center of the shake flask, rotating around the z-axis (see Fig. S2). To exclude the liquid film, liquid contact lines from CFD were extracted at multiple distances from 50 to 4550 µm normal to the shake flask wall (as indicated in Fig. S1A). Instead of determining a Newtonian viscosity for the simulations, as done in Fig. 1, consistency factor and flow behavior index are used in the CFD simulation and shear rates estimated by OpenFOAM. Simulated cases are essentially identical to the simulations shown in Fig. 1, except in the description of the rheological behavior. Simulated conditions: Shaking diameter (d_0_) = 25 mm, surface tension (σ) = 70 mN/m, contact angle (θ) = 20°, temperature (T) = 20°C, consistency factor (*K*) = 104 mPa·s, (*m*) flow behavior index = 0.956
